# Global research trends and emerging frontiers of intratumoral microbiota in cancer immunotherapy: a bibliometric and visualization analysis

**DOI:** 10.3389/fimmu.2026.1865305

**Published:** 2026-07-21

**Authors:** Jiahe Zhou, Heyun Tao, Fuzhu Zou, Huiying Guo, Ruo Ding, Qing Lei, Xiaodie Zhou, Si Mei

**Affiliations:** 1 College of Integrated Chinese and Western Medicine, Hunan University of Chinese Medicine, Changsha, Hunan, China; 2 College of Acupuncture, Massage and Rehabilitation, Hunan University of Chinese Medicine, Changsha, Hunan, China; 3 Faculty of Medicine, Hunan University of Chinese Medicine, Changsha, Hunan, China; 4 College of Chinese Medicine, Hunan University of Chinese Medicine, Changsha, Hunan, China; 5 Department of Physiology, Faculty of Medicine, Hunan University of Chinese Medicine, Changsha, Hunan, China; 6 Department of Biochemistry & Molecular Biology, Cumming School of Medicine, University of Calgary, Calgary, AB, Canada

**Keywords:** bibliometric analysis, cancer immunotherapy, immune checkpoint inhibitors, intratumoral microbiota, tumor immune microenvironment

## Abstract

**Background:**

Intratumoral microbiota, an important component of the tumor microenvironment (TME), have attracted increasing attention in cancer immunotherapy. Emerging evidence links intratumoral microbiota to tumor immune microenvironment (TIME) remodeling, immune cell infiltration, and heterogeneous responses to immune checkpoint inhibitors (ICIs). However, the overall research landscape, knowledge base, and hotspot evolution in this field remain insufficiently characterized. This study aimed to systematically map this field through bibliometric and visualization analyses.

**Methods:**

Publications up to November 8, 2025, were retrieved from the Web of Science Core Collection, Scopus, and PubMed. After screening, deduplication, and data standardization, bibliometric analyses were performed using R, VOSviewer, CiteSpace, and Scimago Graphica to examine publication trends, collaboration networks, knowledge bases, and keyword evolution.

**Results:**

A total of 245 publications were included, comprising 141 original articles and 104 reviews. Since the first publication appeared in 2017, the field has grown exponentially, with a compound annual growth rate (CAGR) of 63.1% from 2017 to 2024. China ranked first in publication output, followed by the United States, while the United States occupied a more central position in total citations and international collaboration. *Frontiers in Immunology* was the most productive journal, whereas *Science*, *Cell*, and *Nature* constituted the major co-cited knowledge base, with 1,172, 729, and 670 co-citations, respectively. Keyword analysis showed that “intratumoral microbiota” was the most frequent term (90 occurrences), with excellent clustering quality (modularity Q = 0.6963; silhouette S = 0.9351). Research hotspots have gradually shifted from early explorations of gut microbiota, CD8^+^ T cells, and immune mechanisms toward immunotherapy resistance, microbial biomarkers, and microbiota-targeted interventions, including engineered bacteria, extracellular vesicles, and fecal microbiota transplantation.

**Conclusion:**

Research on intratumoral microbiota in cancer immunotherapy has rapidly developed into a distinct interdisciplinary field. Current hotspots are moving from mechanistic exploration toward response prediction and translational intervention. Future studies should prioritize standardized detection, spatial and multi-omics validation, and multicenter prospective evaluation to support the clinical translation of microbiota-based biomarkers and therapeutic strategies.

## 1 Introduction

Intratumoral microbiota has attracted increasing attention in recent years as an important component of the tumor microenvironment (TME). For a long time, tumor tissues were widely assumed to be sterile because of technical limitations in microbial detection. However, with advances in high-throughput sequencing, spatial omics, and multiplex imaging technologies, accumulating evidence has demonstrated the presence of metabolically active bacterial communities in a variety of solid tumors. These bacteria are primarily localized within tumor cells and stromal regions and display tumor type-specific compositional patterns (1). Previous studies have linked intratumoral microbiota to remodeling of the tumor immune microenvironment (TIME), including changes in immune cell infiltration, immune cell function, and antitumor immune responses (2). Unlike the gut microbiota, which mainly influences tumors through systemic immune regulation, the intratumoral microbiota reside directly within the local tumor niche and are therefore more likely to shape tumor type-specific immunosuppressive states, local inflammatory responses, and heterogeneity in therapeutic outcomes. Accordingly, intratumoral microbiota should not be viewed merely as an extension of the broader microbiota–tumor research framework, but as an emerging field centered on tumor-local spatial ecology and immune regulation.

Cancer immunotherapy, particularly immune checkpoint inhibitors (ICIs), has substantially improved outcomes in multiple solid tumors. Nevertheless, the overall response rate remains limited, and both primary and acquired resistance are common (3, 4). In recent years, accumulating studies have linked intratumoral microbiota to variation in ICI response, potentially through associations with the local immune ecosystem, immune checkpoint-related signaling, and effector T-cell states (5). At the same time, research on intratumoral microbiota has evolved from early descriptive studies of its presence and associations with tumor phenotypes toward deeper investigations of underlying mechanisms, treatment response prediction, and therapeutic intervention (6–9).

Despite this rapid growth, research in this field remains highly fragmented. First, relevant studies span multiple disciplines, including microbiology, immunology, oncology, and translational medicine, and involve diverse tumor types, microbial levels of analysis, and detection platforms. Second, most existing reviews focus on mechanistic interpretation or on the roles of specific microorganisms or tumor types, whereas systematic quantitative analyses of the field’s developmental trajectory, knowledge base, international collaboration patterns, and hotspot evolution remain scarce. For an emerging interdisciplinary field with both mechanistic depth and translational potential, traditional narrative reviews alone are no longer sufficient to fully capture its knowledge structure or developmental trajectory.

As a data-driven approach, bibliometric analysis can systematically characterize the developmental trajectory, knowledge structure, and research hotspots of a field by analyzing multidimensional information such as publication trends, collaboration among countries and institutions, author networks, journal co-citation patterns, and keyword evolution (10). Therefore, a systematic bibliometric and visualization analysis of intratumoral microbiota in cancer immunotherapy is needed not only to clarify the overall research landscape of the field, but also to identify directions with sustained academic influence and translational potential. To this end, the present study integrated relevant publications retrieved from the Web of Science Core Collection (WoSCC), Scopus, and PubMed databases, and combined R, VOSviewer, CiteSpace, and Scimago Graphica to systematically analyze publication trends, international collaboration, institutional and author cooperation networks, journal co-citation patterns, and keyword co-occurrence and burst dynamics. Through this approach, we sought to provide a comprehensive overview of the current landscape, developmental trajectory, and research hotspots of the field, thereby informing future basic research, microbial biomarker development, and translational efforts in precision cancer immunotherapy.

## 2 Materials and methods

### 2.1 Data sources and search strategy

This study followed the PRISMA 2020 guidelines for literature retrieval, screening, and data extraction to enhance the transparency and reproducibility of the study process (11). To maximize coverage of studies on intratumoral microbiota in cancer immunotherapy, three major databases were used as primary data sources: WoSCC, Scopus, and PubMed. All searches were conducted within the same time window to minimize potential bias arising from database updates, and the final search date was November 8, 2025.

The search strategy was built around two core concepts, “intratumoral microbiota” and “cancer immunotherapy,” and combined term variants, Boolean operators, and wildcards. To improve relevance and reduce retrieval of non-core records, the search scope was restricted to the title, abstract, and author keyword fields, while automatically generated indexing terms were excluded. Specifically, the WoSCC search was limited to the TI, AB, and AK fields; Scopus searches covered the TITLE, ABS, and AUTHKEY fields; and PubMed search was restricted to the Title and Abstract fields. Only English-language publications were included, and document types were limited to articles and reviews. The full search strategies are provided in **Supplementary Material 1**.

### 2.2 Study screening and data preprocessing

A total of 651 records were initially retrieved, including 207 from WoSCC, 226 from Scopus, and 218 from PubMed.

The following inclusion and exclusion criteria were established prior to literature screening. The inclusion criteria were as follows: (1) original research articles or review articles; (2) publications written in English; and (3) studies focusing on the relationship between intratumoral microbiota and cancer immunotherapy, including studies related to ICIs, antitumor immunity, immunotherapy response or resistance, and immunotherapy-associated TIME remodeling.

The exclusion criteria were as follows: (1) non-peer-reviewed document types, including conference abstracts, conference proceedings, editorials, letters, comments, notes, book chapters, and academic theses; (2) studies focusing exclusively on gut microbiota without involving intratumoral microbiota; and (3) duplicate records retrieved from multiple databases; (4) records lacking essential bibliographic information when they could not be reliably standardized for bibliometric analysis.

**Figure 1:**
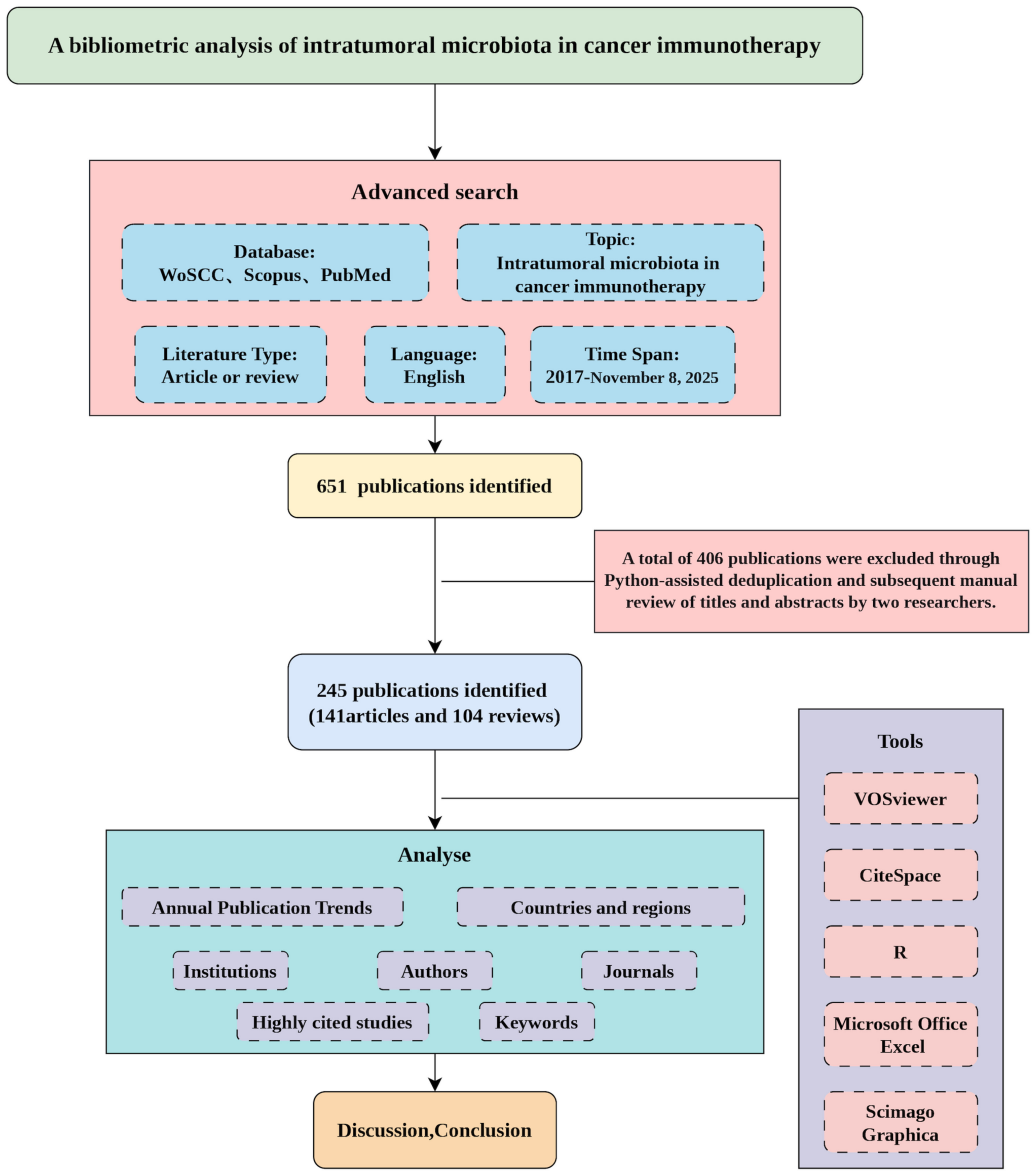
Flowchart of the literature screening process.

Literature screening was independently performed by two researchers based primarily on titles, abstracts, and keywords to assess relevance to the study topic. Any disagreements were resolved through consultation with a third researcher. The detailed screening process is shown in **Figure 1**.

After duplicate and irrelevant records were removed, bibliographic data were exported in plain-text and CSV formats, including titles, authors, affiliations, countries, publication years, abstracts, keywords, and references. To harmonize data structures across databases, Python (version 3.11) was used to convert the CSV files exported from Scopus into a plain-text format consistent with the records from WoSCC and PubMed.

All records were then standardized using Python (version 3.11). The main preprocessing steps were as follows:

1. DOI-based deduplication;
2. removal of records with “[Anonymous]” in the author field;
3. exclusion of entries without substantive institutional attributes, such as “Egyptian Knowledge Bank (EKB)”;
4. merging and standardization of duplicate or variant institutional names, country/region names, author names, and keywords, for example, standardizing “U.S.A.” and “United States” as “USA”, and “intratumoral microbiome” as “intratumoral microbiota”, to ensure consistent counting and cross-database analysis.

After screening and data cleaning, 245 publications were ultimately included in the bibliometric analyses.

### 2.3 Bibliometric analysis and visualization

To systematically characterize the research landscape, collaboration patterns, knowledge base, and research hotspots of this field, we adopted a multi-software analytical framework in which each tool was used according to its respective strengths.

First, basic bibliometric analyses were performed using the bibliometrix package in R (version 4.5.1), including analyses of annual publication trends, national research output, and international collaboration patterns. Trend plots were generated using the ggplot2 package. To quantify the temporal growth pattern of this field, an exponential growth model was further fitted to annual publication output from 2017 to 2024 using the nls() function.

Second, VOSviewer was used to construct country- and institution-level collaboration networks as well as journal co-citation networks. In the collaboration maps, node size was proportional to publication output, whereas link thickness reflected collaboration strength. In the journal co-citation network, node size represented co-citation frequency, whereas link thickness indicated the strength of co-citation relationships. Scimago Graphica (version 1.0.52) was used to generate the global country collaboration map showing the geographic distribution of international cooperation.

Finally, CiteSpace was employed to analyze the field’s knowledge base and research frontiers, including reference co-citation, keyword clustering, and burst detection. Keyword cluster labels were generated using the log-likelihood ratio (LLR) algorithm. The quality of keyword clustering was evaluated using modularity (Q value) and silhouette (S value), which reflect clustering structure and internal consistency, respectively. The main CiteSpace parameters were set as follows: time slicing from 2017 to 2025 with 1 year per slice; node selection based on the g-index (k = 25); and network pruning using pathfinder, pruning sliced networks, and pruning the merged network. These settings were retained in the generated maps or corresponding figure legends.

## 3 Results

After systematic retrieval, screening, deduplication, and data standardization, 245 publications were ultimately included in this study (**Figure 1**), comprising 141 original articles (57.55%) and 104 reviews (42.45%). These publications were analyzed to characterize the research landscape, knowledge base, and hotspot evolution in the field of intratumoral microbiota and cancer immunotherapy from the perspectives of annual publication trends, country- and institution-level collaboration, author and journal distribution, co-citation networks, and keyword dynamics.

### 3.1 Publication trends

**Figure 2:**
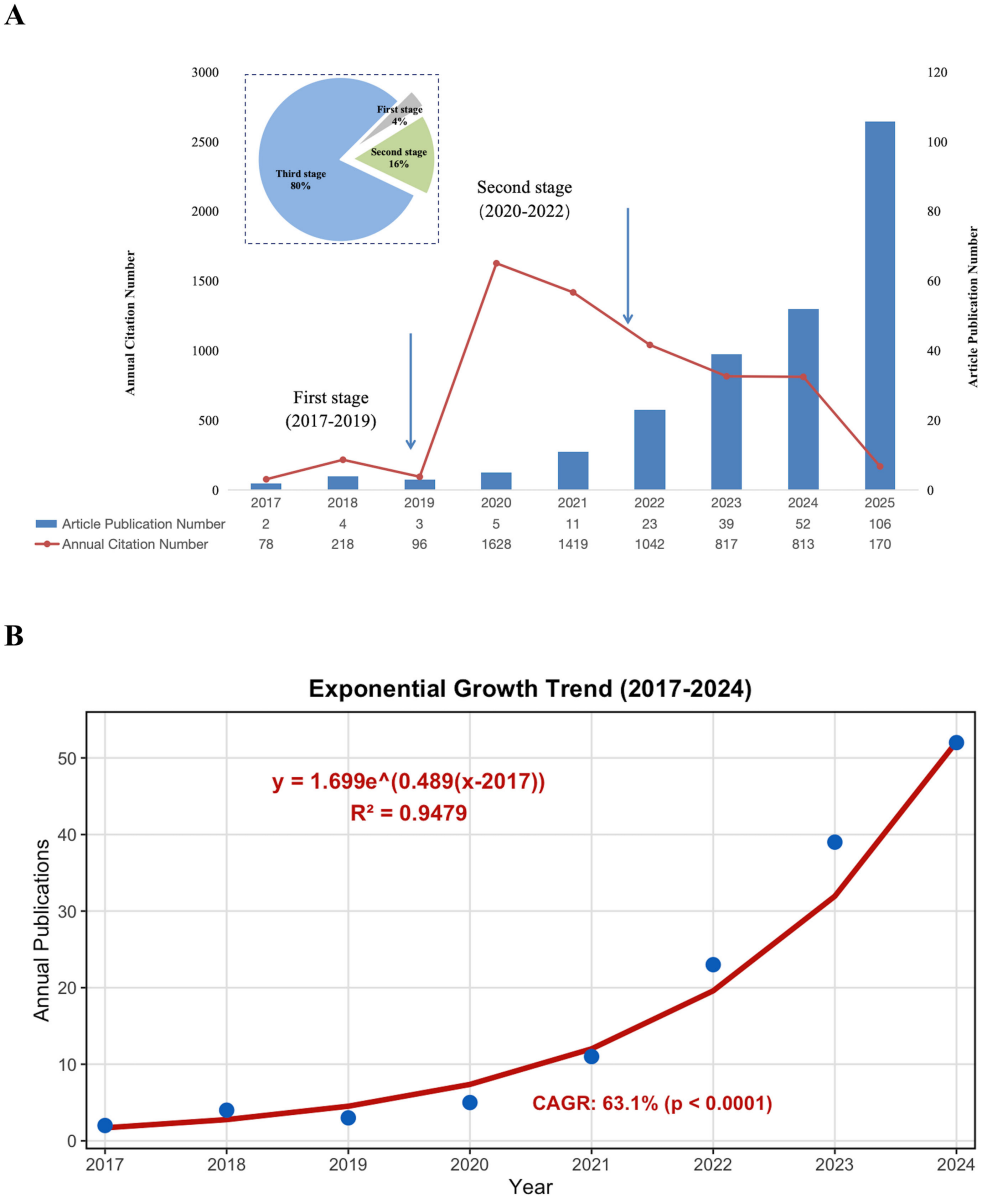
Temporal trends in publication and citation growth. **(A)** Annual publications (blue bars) and citation counts (red line) from 2017 to 2025. **(B)** Exponential growth model fitting of annual publications from 2017 to 2025.

The first publication relevant to this field appeared in 2017. Since then, annual publication output has increased steadily, with a marked acceleration after 2021 (**Figure 2A**). Annual publication output increased exponentially from 2017 to 2024, with a compound annual growth rate (CAGR) of 63.1% (*p* < 0.0001) (**Figure 2B**) and peaked in 2024. It should be noted that the publication count for 2025 was calculated only through November 8, 2025, and may therefore be underestimated. Overall, research on intratumoral microbiota in cancer immunotherapy has rapidly progressed from an initial exploratory stage to accelerated expansion, suggesting that the field remains in an active growth phase and has not yet reached a stable plateau.

### 3.2 Country/region analysis

At the country level, research on intratumoral microbiota in cancer immunotherapy showed a clear global distribution pattern, with China and the United States emerging as the two major contributors. Overall, China led in publication output, whereas the United States occupied a more central position in terms of total citations and international collaboration. **Table 1** summarizes the publication output, total citations, and total link strength of the 10 most productive countries or regions. China ranked first with 152 publications, accounting for 62.04% of the total output, indicating a substantial productivity advantage. The United States ranked second with 52 publications, but exceeded China in total citation (N = 3,740) and average impact, suggesting greater academic influence and international visibility.

**Table 1:** Top 10 countries/regions by publication output.

Rank	Country	Publications	Citations	Average citations	Total link strength
1	China	152	1840	12.11	15
2	USA	52	3740	71.92	29
3	Italy	11	1656	150.55	10
4	France	7	1427	203.86	6
5	Germany	7	326	46.57	5
6	Israel	5	2268	453.60	8
7	Netherlands	5	1434	286.80	8
8	Japan	5	31	6.20	0
9	Singapore	4	143	35.75	5
10	India	4	46	11.50	5

**Figure 3:**
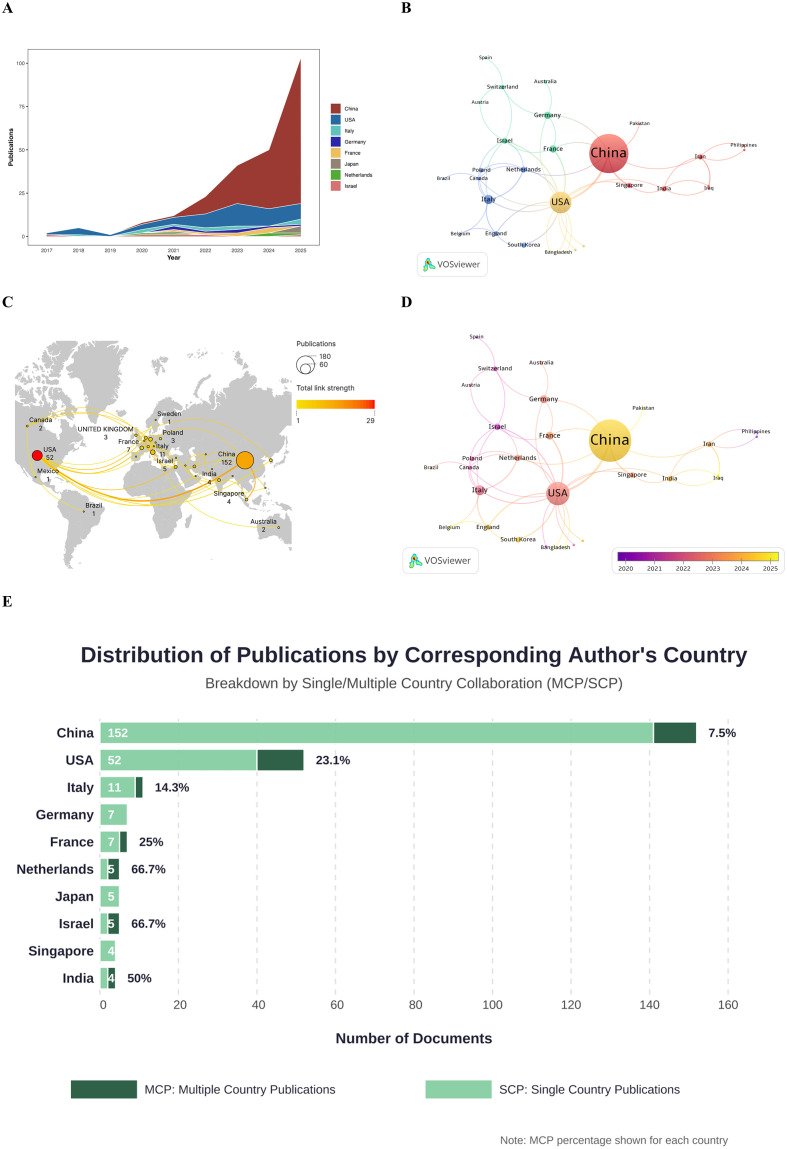
Country-level publication trends and collaboration patterns. **(A)** Annual publication trends of major countries from 2017 to 2025. **(B)** Network visualization of international collaboration among countries. Node size is proportional to total publication output. Edge thickness represents collaboration strength. Node colors indicate distinct collaboration clusters identified. **(C)** Global map of international research collaboration. Circle size reflects publications. Line color intensity represents total link strength, with darker colors indicating stronger collaborative relationships. **(D)** Temporal overlay of the country collaboration network. Node color gradient from purple (earlier average publication year) to yellow (later average publication year). **(E)** International co-authorship patterns of corresponding authors by country.

**Figure 3A** illustrates annual publication trends among the major contributing countries. From a temporal perspective, early research was primarily driven by the United States, Israel, and Italy. Since 2020, China has shown a rapid increase in output and has remained the leading contributor thereafter. Meanwhile, publication output from the United States and Italy also increased steadily. Further analysis of international collaboration patterns showed that China and the United States were the two main hubs in the collaboration network (**Figure 3B**), with the strongest bilateral collaboration observed between them. China maintained relatively close collaborations with the United States, France, Singapore, and Germany. In addition to its strong collaboration with China, the United States also collaborated frequently with Italy, the Netherlands, and the United Kingdom. The United States showed the darkest node color and the broadest range of connections, indicating higher centrality and stronger connectivity in the global collaboration network (**Figure 3C**). Dynamic visualization of country collaboration patterns (**Figure 3D**) further showed that the United States dominated the early phase of development in this field, whereas China became increasingly active in later years and gradually emerged as a major research center. Corresponding-author analysis at the country level (**Figure 3E**) revealed that China had the highest number of corresponding-author publications (152 papers), but a relatively low multiple-country publication (MCP) ratio of only 7.5%, suggesting that its research output was still mainly driven by domestic teams. By contrast, the United States and France exhibited a more balanced pattern of domestic and international collaboration (MCP > 23%). In addition, Germany, Japan, and Singapore showed no multiple-country corresponding-author publications (MCP = 0). Taken together, **Table 1** and **Figure 3** indicate that research in this field has entered an internationalized stage, although the global collaboration network still has considerable room for further integration and expansion.

### 3.3 Institution analysis

Institution-level analysis showed that the field has developed an institutional landscape dominated by Chinese institutions, while also involving several internationally influential centers. Among these institutions, The University of Texas MD Anderson Cancer Center stood out in terms of publication output, citation impact, and collaboration network connectivity. **Table 2** lists the publication output, total citations, and total link strength of the 10 most productive institutions. Nine of these institutions were based in China. The three most productive institutions each published seven papers: The University of Texas MD Anderson Cancer Center, Sun Yat-sen University, and Nanjing Medical University. In terms of total citations, The University of Texas MD Anderson Cancer Center ranked first (N = 2,765), followed by the Chinese Academy of Sciences (N = 338) and Sun Yat-sen University (N = 114). Overall, The University of Texas MD Anderson Cancer Center ranked among the leading institutions in publication output, citation impact, and total link strength, indicating strong academic influence and network connectivity.

**Table 2:** Top 10 institutions by publication output.

Rank	Institution	Country	Publications	Citations	Total link strength	Average citations
1	The University of Texas MD Anderson Cancer Center	America	7	2765	10	395.00
2	Sun Yat-sen University	China	7	114	2	16.29
3	Nanjing Medical University	China	7	105	15	15.00
4	Chinese Academy of Sciences	China	6	338	14	56.33
5	Soochow University	China	6	106	6	17.67
6	Geneis Beijing Co Ltd	China	6	14	10	2.33
7	Zhengzhou University	China	5	105	3	21.00
8	Yangzhou University	China	5	64	3	12.80
9	Dalian Medical University	China	5	46	4	9.20
10	Southern Medical University	China	5	36	5	7.20

**Figure 4:**
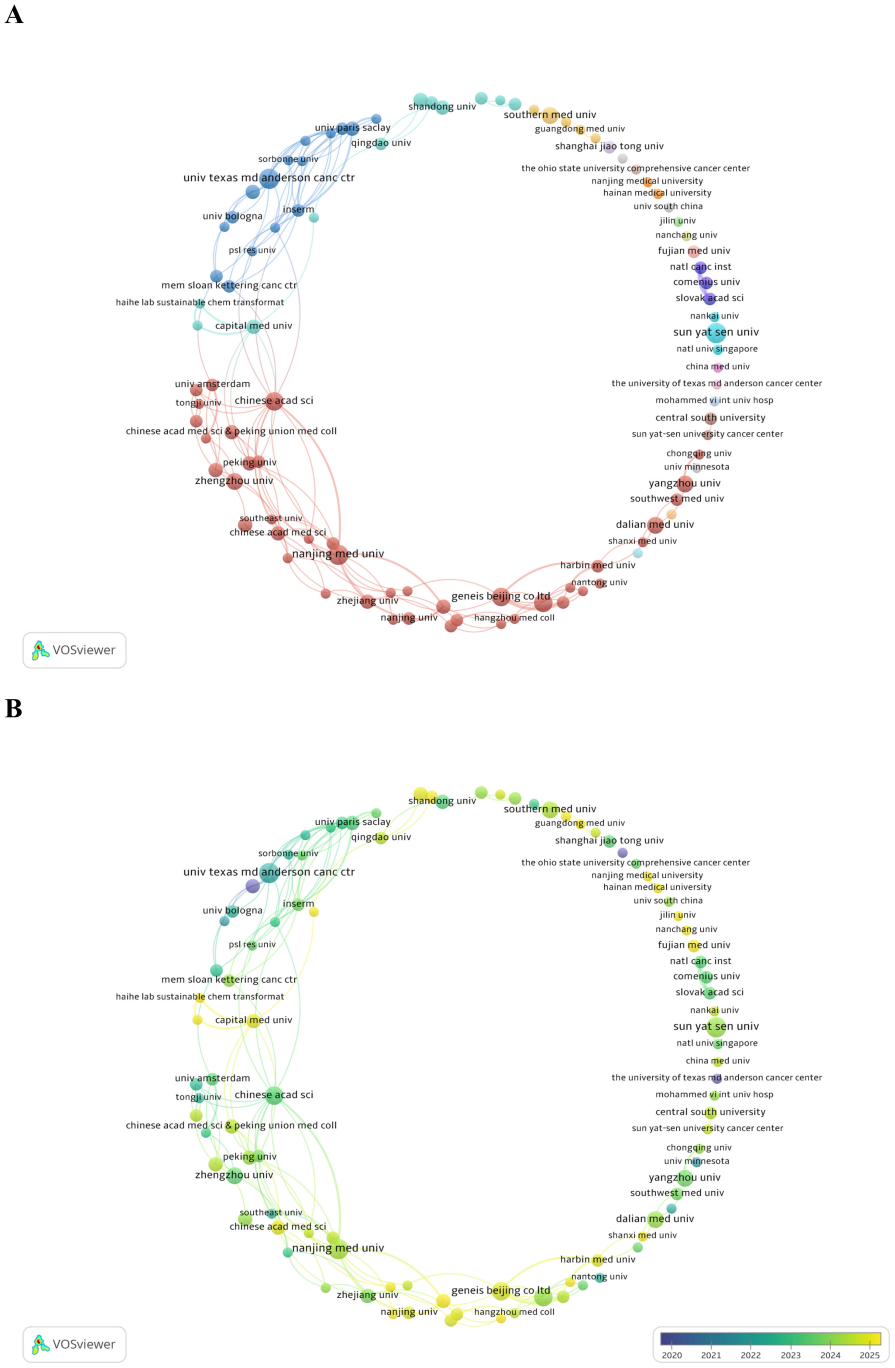
Institutional collaboration patterns and temporal evolution. **(A)** Collaboration network of institutions with at least two publications. Node size is proportional to publication output; edge thickness represents collaboration strength. Node colors indicate distinct collaboration clusters. **(B)** Temporal overlay of the institutional collaboration network. Node color gradient from blue (earlier average publication year) to yellow (later average publication year).

Institutions with at least two publications were further selected to construct an institutional collaboration network (**Figure 4A**). The resulting network showed a multicenter and multilevel collaboration structure. Several major Chinese clusters were evident, centered on institutions such as Nanjing Medical University, the Chinese Academy of Sciences, and Zhejiang University. These institutions were closely connected to one another and were also linked to international centers such as The University of Texas MD Anderson Cancer Center, Université Paris-Saclay, and the Weizmann Institute of Science. In addition to the core clusters, several smaller regional subclusters were identified, including groups centered on Dalian Medical University and Harbin Medical University. Meanwhile, institutions such as Sun Yat-sen University and Shanghai Jiao Tong University were not fully embedded in the main collaboration clusters, but still appeared as relatively independent and important nodes in the network.

Overlay visualization of temporal activity (**Figure 4B**) further revealed the temporal evolution of institutional contributions. Early research was primarily led by international institutions such as the Weizmann Institute of Science and The University of Texas MD Anderson Cancer Center. In later years, Chinese institutions such as Nanjing Medical University, Zhejiang University, and Sun Yat-sen University became increasingly active. In addition, emerging institutions such as Nanjing University and Hangzhou Medical College also entered the collaboration network. Overall, the institutional center of activity in this field has gradually shifted from early dominance by a limited number of international institutions to broader and sustained participation by Chinese institutions.

### 3.4 Author analysis

Author-level analysis showed that the collaboration network in this field remains relatively fragmented, with only a small number of highly influential scholars serving as bridges between different research clusters. **Table 3** lists the 10 most productive authors ranked by publication output, together with their citation counts. These 10 authors collectively published 39 papers and received 3,024 citations. Among them, five were from China, three from Slovakia, and one each from the United States and France. Notably, the three Slovak authors had identical publication and citation counts, suggesting that their contributions largely came from a stable collaborative team. In terms of academic impact, Jennifer A. Wargo from The University of Texas MD Anderson Cancer Center ranked first with 1,275 citations. In terms of productivity, Ji Lei (Geneis Beijing Co Ltd) ranked first with six publications, followed by Jennifer A. Wargo and Tang Dong (Yangzhou University), each with five publications.

**Table 3:** Top 10 authors by publication output.

Rank	Author	Publications	Country	Institution	Citations	Average citations	Total link strength
1	Ji, Lei	6	China	Geneis Beijing Co Ltd	14	2.33	8
2	Jennifer A. Wargo	5	USA	The University of Texas MD Anderson Cancer Center	1275	255.00	6
3	Tang, Dong	5	China	Yangzhou University	64	12.80	12
4	Zitvogel, Laurence	4	France	Universite Paris Saclay	1269	317.25	10
5	Wang, Qi	4	China	Jiangsu University	48	12.00	1
6	Ciernikova, Sona	3	Slovakia	Slovak Academy of Sciences	84	28.00	6
7	Mego, Michal	3	Slovakia	Comenius University Bratislava	84	28.00	6
8	Sevcikova, Aneta	3	Slovakia	Slovak Academy of Sciences	84	28.00	6
9	Wang, Daorong	3	China	Yangzhou University	51	17.00	11
10	Zhang, Wenjie	3	China	Yangzhou University	51	17.00	11

**Figure 5:**
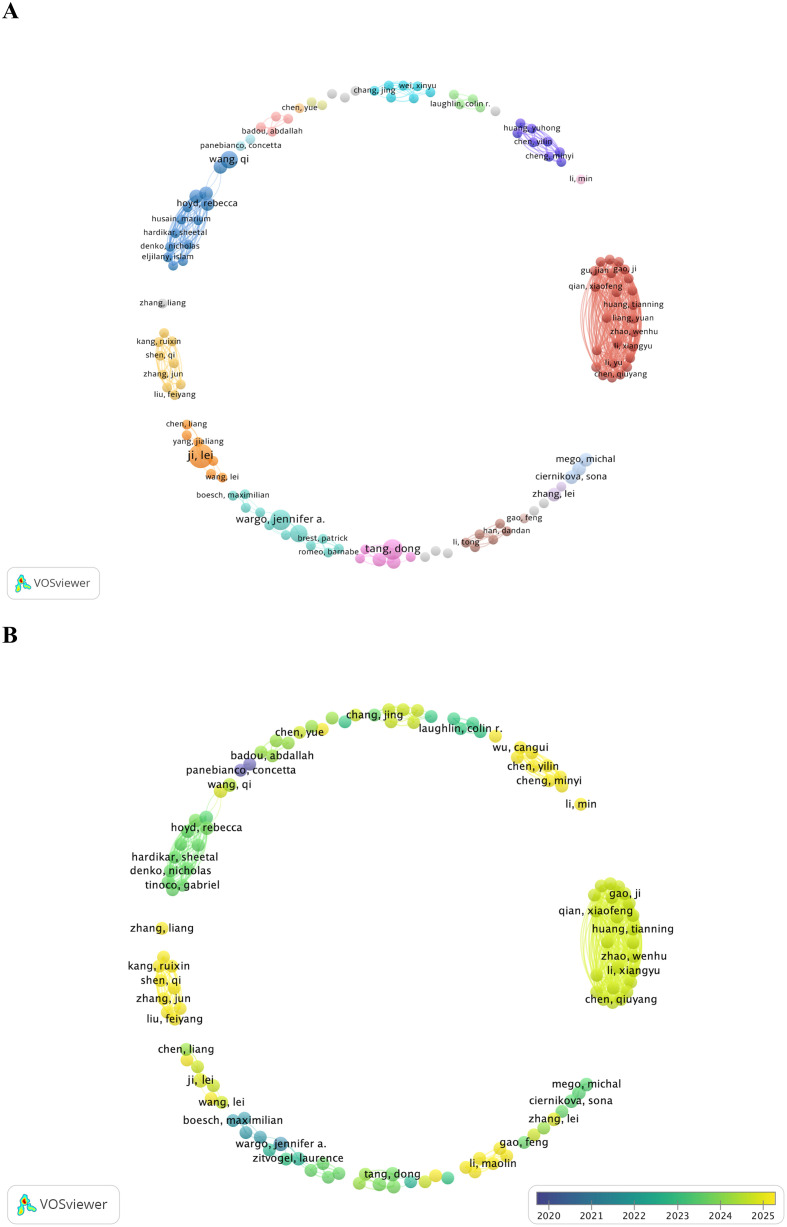
Author collaboration patterns and temporal evolution. **(A)** Collaboration network of core authors with at least two publications. Node size is proportional to publication output. Edge thickness represents collaboration strength. Node colors indicate distinct collaboration clusters identified. **(B)** Temporal overlay of the author collaboration network. Node color gradient from blue (earlier average publication year) to yellow (later average publication year).

According to Price’s law, authors with at least two publications were defined as core authors. Based on this criterion, 123 core authors were identified and used to construct the author collaboration network (**Figure 5A**). The resulting network indicated that collaboration among authors was generally loose and that a highly integrated large-scale collaboration network has not yet formed. Specifically, Chinese scholars formed several relatively independent collaboration subclusters centered on authors such as Tang Dong, Wang Daorong, and Ji Lei. The Slovak group was represented by a tightly connected small team centered on Mego Michal and Ciernikova Sona. Cross-national collaboration was maintained largely by a few highly influential scholars, such as Jennifer A. Wargo from the United States and Laurence Zitvogel from France. Overlay visualization of temporal activity (**Figure 5B**) further illustrated changes in author activity over time. Early studies (2020–2021) were primarily driven by scholars from the United States and France, whereas in later years (2022–2024), Chinese scholars became markedly more active and gradually emerged as the main driving force in this field. Overall, the author-level landscape has evolved from early dominance by a small number of highly influential international researchers to broader participation by Chinese research teams.

### 3.5 Analysis of journals and co-cited journals

Journal analysis showed that studies in this field were published mainly in immunology-, oncology-, and molecular biology–related journals, whereas the co-citation structure was centered on high-impact multidisciplinary journals such as *Science*, *Cell*, and *Nature*, which together formed the core knowledge base of the field. **Table 4** lists the 10 source journals with the highest publication output. *Frontiers in Immunology*, published in Switzerland, ranked first with 17 publications (6.94%), followed by Cancers with 9 publications (3.67%). In terms of average citations per article, International Journal of Molecular Sciences (45.60) and Advanced Science (54.00), despite their relatively low publication output, showed strong citation impact. Although Science published only two relevant papers, it received 2,189 total citations, corresponding to an average of 1,094.5 citations per paper. Overall, 70% of the 10 most productive source journals were ranked in Journal Citation Reports (JCR) Q1, indicating that most studies in this field have been published in high-impact journals.

**Table 4:** Top 10 journals by publication output and co-citation frequency.

Rank	Top 10 source journals								Top 10 co-cited journals				
	Source	Country	Documents	Citations	Total link strength	Average citations	IF	JCR(2025)	Source	Co-citations	Total link strength	IF	JCR(2025)
1	Frontiers in Immunology	Switzerland	17	183	57	10.76	5.9	Q1	Science	1172	35251	56.9	Q1
2	Cancers	Switzerland	9	73	15	8.11	4.5	Q2	Cell	729	24241	64.5	Q1
3	Journal of Translational Medicine	England	6	25	29	4.17	7.4	Q1	Nature	670	23238	64.8	Q1
4	International Journal of Molecular Sciences	Switzerland	5	228	10	45.60	4.9	Q1	Nature Communications	442	14468	16.6	Q1
5	Frontiers in Oncology	Switzerland	5	16	17	3.20	3.3	Q2	Frontiers in Immunology	391	10483	5.9	Q1
6	Biomaterials	Netherlands	5	4	7	0.80	12.8	Q1	Nature Medicine	349	13086	82.9	Q1
7	Seminars in Cancer Biology	England	4	64	6	16.00	15.7	Q1	Cancer Cell	325	11535	50.3	Q1
8	Microbiology Spectrum	America	4	36	15	9.00	3.8	Q2	Gut	318	12158	25.8	Q1
9	Advanced Science	America	3	162	6	54.00	14.1	Q1	Scientific Reports	271	9538	3.9	Q1
10	Cancer Research	America	3	88	15	29.33	16.6	Q1	Cell Host & Microbe	264	10785	18.7	Q1

**Figure 6:**
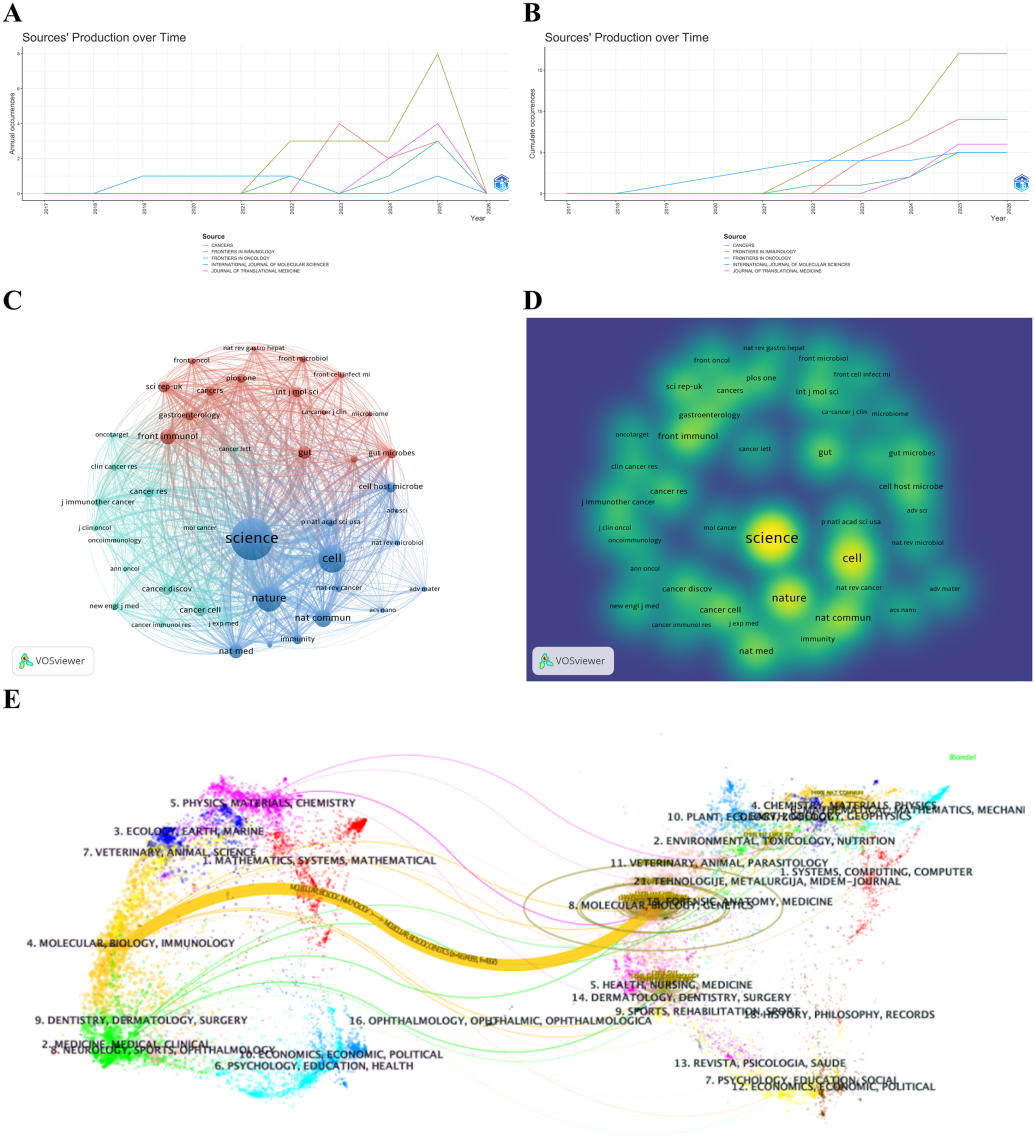
Journal publication trends and co-citation analysis. **(A)** Annual publication trends of core journals. **(B)** Cumulative publication trends of core journals. **(C)** Co-citation network of journals with at least 100 co-citations (N = 43). Node size is proportional to co-citation frequency. Edge thickness represents co-citation strength. Node colors indicate distinct co-citation clusters identified. **(D)** Density visualization of co-cited journals. Higher density (yellow) indicates stronger co-citation relationships, concentrated around *Nature*, *Science*, and *Cell*. **(E)** Dual-map overlay of journals related to the publications. Colored paths indicate citation flows between citing journals (left) and cited journals (right).

The geographic distribution of publishers was diverse, with four based in Switzerland, three in the United States, two in the United Kingdom, and one in the Netherlands. **Figures 6A, B** present the annual and cumulative publication trends of the major journals, respectively. The results showed that journals such as Frontiers in Immunology and International Journal of Molecular Sciences have published studies in this field consistently in recent years, reflecting their sustained activity in this topic area. At the same time, different journals exhibited distinct growth trajectories. International Journal of Molecular Sciences began publishing related studies relatively early, followed by a gradual increase in output from Frontiers in Immunology, whereas journals such as Frontiers in Oncology and Journal of Translational Medicine showed more pronounced growth after 2023. This temporal pattern suggests that research outputs in this field were initially scattered across molecular and basic research journals, but gradually expanded into immunology- and translational medicine-oriented journals.

Co-cited journal analysis further clarified the knowledge base of the field. **Table 4** also lists the 10 most highly co-cited journals, all of which were cited more than 250 times, with three receiving more than 600 co-citations. *Science* ranked first (N = 1,172), followed by *Cell* (N = 729) and Nature (N = 670), and together these journals constituted the most important knowledge sources in the field. In addition, Nature Communications, Frontiers in Immunology, and Nature Medicine also showed high co-citation frequencies, indicating their important roles in the field’s knowledge system.

The co-cited journal network identified three major clusters (**Figure 6C**). The first cluster, represented by *Science*, *Nature*, *Cell*, *Nature Communications*, and *Nature Medicine*, reflected the core knowledge base of fundamental immunology and microbial mechanism studies. The second cluster, including Frontiers in Microbiology, Gut, Gastroenterology, Scientific Reports, and Microbiome, reflected the literature base of gut microbiota, gastroenterology, and related clinical studies. The third cluster, represented by Cancer Cell, Cancer Discovery, Journal for ImmunoTherapy of Cancer, and Clinical Cancer Research, corresponded to cancer immunotherapy and translational application studies. Density visualization of co-cited journals (**Figure 6D**) further showed that the highest-density area was centered on Nature, Science, and Cell, indicating that high-frequency co-citation relationships in this field were concentrated around basic studies of immunity and microbial mechanisms. The dual-map overlay (**Figure 6E**) further showed that the main citation paths between citing and cited journals were concentrated in the “4. MOLECULAR, BIOLOGY, IMMUNOLOGY” area, indicating that both the knowledge base and research output of this field are primarily rooted in molecular biology and immunology.

### 3.6 Citation analysis

Citation analysis showed that highly cited references and burst references together constitute the main knowledge base of the field. However, representative studies directly focused on intratumoral microbiota remain limited in number, whereas many highly influential burst references still originate from the broader field of microbiota and cancer immunotherapy. **Table 5** lists the 10 most co-cited references, among which basic research articles predominated. The most cited reference was the 2020 *Science* article by Deborah Nejman et al., entitled The human tumor microbiome is composed of tumor type-specific intracellular bacteria. This study systematically demonstrated the existence of tumor type-specific intracellular bacteria in human tumors and provided important foundational evidence for subsequent research on intratumoral microbiota.

**Table 5:** Top 10 most co-cited references.

Rank	References	Journal	Author	Corresponding author	Year	Co-citations	Type	Description
1	The human tumor microbiome is composed of tumor type-specific intracellular bacteria DOI:10.1126/science.aay9189	Science	Deborah Nejman	Ravid Straussman	2020	107	Research	The human tumor microbiome is composed of tumor type-specific intracellular bacteria that influence the tumor microenvironment, progression, and therapeutic response.
2	Tumor-resident intracellular microbiota promotes metastatic colonization in breast cancer DOI:10.1016/j.cell.2022.02.027	Cell	Aikun Fu	Shang Cai	2022	73	Research	Tumor-resident intracellular bacteria promote metastatic colonization in breast cancer by remodeling the cytoskeleton and enhancing cancer cell migration.
3	Tumor Microbiome Diversity and Composition Influence Pancreatic Cancer Outcomes DOI:10.1016/j.cell.2019.07.008	Cell	Erick Riquelme	Florencia McAllister	2019	59	Research	Pancreatic cancer microbiome diversity and composition correlate with patient survival and serve as a potential prognostic marker.
4	Effect of the intratumoral microbiota on spatial and cellular heterogeneity in cancer DOI:10.1038/s41586-022-05435-0	Nature	Jorge Luis Galeano Niño	Susan Bullman	2022	58	Research	Intratumoral microbiota shape spatial and cellular heterogeneity, creating immunosuppressive regions associated with T-cell exclusion.
5	Microbiome analyses of blood and tissues suggest cancer diagnostic approach DOI:10.1038/s41586-020-2095-1	Nature	Gregory D Poore	Rob Knight	2020	46	Research	Blood and tissue microbiome analyses enable cancer detection using microbial DNA signatures.
6	The microbiome and human cancer DOI:10.1126/science.abc4552	Science	Gregory D Sepich-Poore	Rob Knight	2021	45	Review	The microbiome contributes to carcinogenesis, diagnosis, and therapy in human cancer.
7	Breast cancer colonization by Fusobacterium nucleatum accelerates tumor growth and metastatic progression DOI:10.1038/s41467-020-16967-2	Nature Communications	Lishay Parhi	Yardena Samuels	2020	43	Research	Fusobacterium nucleatum colonization accelerates breast cancer growth and modulates immune responses.
8	Identification of bacteria-derived HLA-bound peptides in melanoma DOI:10.1038/s41586-021-03368-8	Nature	Shelly Kalaora	Gilad Bachrach	2021	43	Research	Bacteria-derived HLA-bound peptides in melanoma are recognized by T cells, influencing immune surveillance and therapeutic response.
9	Gut microbiome modulates response to anti-CD19 CAR T cell therapy in B cell malignancies DOI:10.1084/jem.20192282	Journal of Experimental Medicine	Yaoyao Shi	Yang-Xin Fu	2020	40	Research	Gut microbiome modulates anti-CD19 CAR T-cell therapy responses in B-cell malignancies.
10	Intratumoral microbiota: roles in cancer initiation, development and therapeutic efficacy DOI:10.1038/s41392-022-01304-4	Signal Transduction and Targeted Therapy	Li Yang	Yi Zhang	2023	39	Review	Intratumoral microbiota play roles in cancer initiation, progression, and therapeutic efficacy.

**Figure 7:**
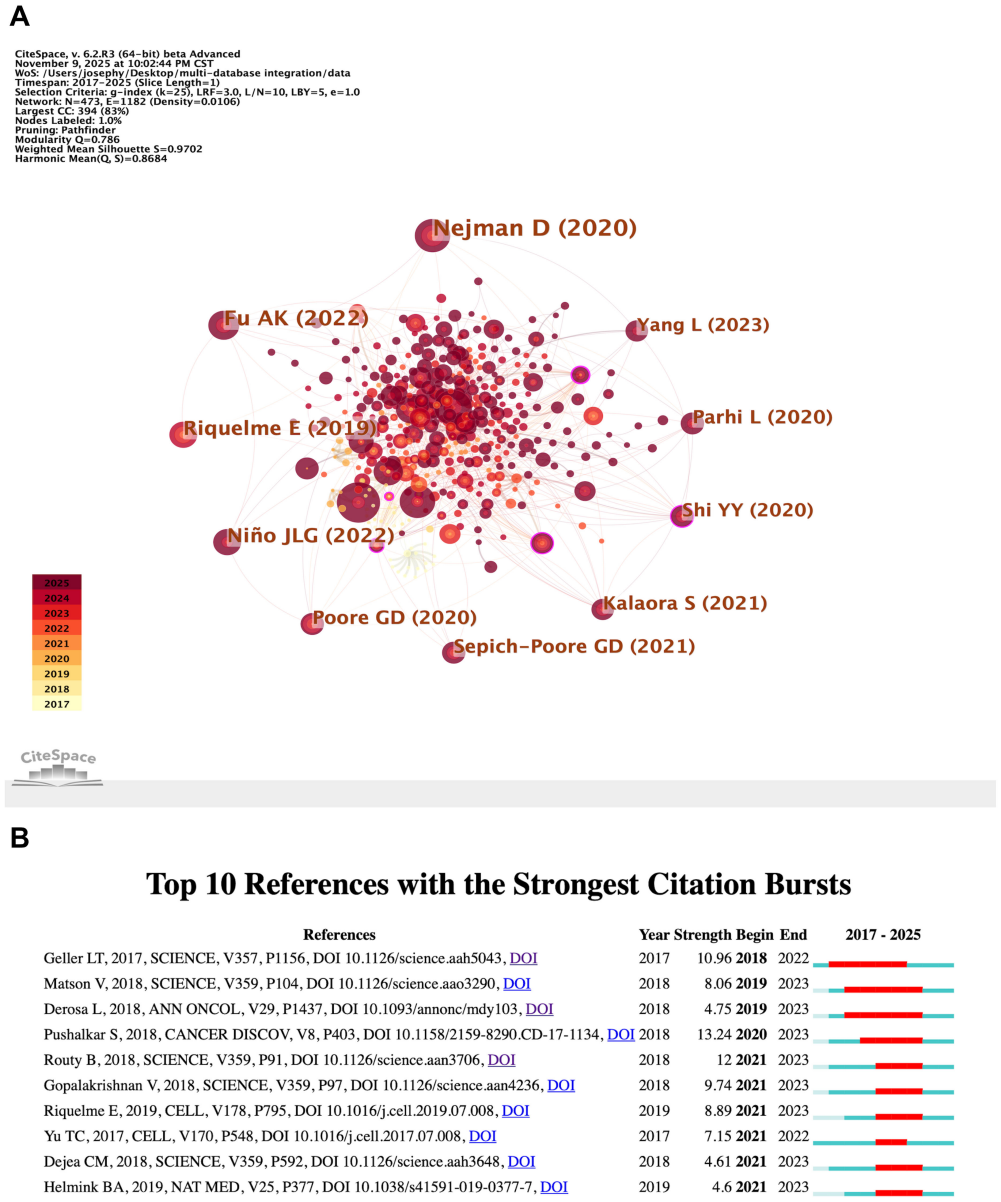
Reference co-citation network and citation burst analysis. **(A)** Co-citation network of the top 10 most frequently co-cited references. Node size is proportional to co-citation frequency. **(B)** Citation burst analysis of the top 10 references with the strongest citation bursts. Red bars indicate burst periods; blue line represents the timeline (2017-2025).

**Figure 7A** presents the co-cited reference network. The results showed that a limited number of highly influential studies formed the core nodes of the co-citation network, indicating that the current knowledge base of the field is still supported mainly by a relatively small number of foundational studies. **Figure 7B** shows the reference burst analysis. Among all burst references, only a small number focused directly on intratumoral microbiota itself. The strongest burst was observed for the 2018 Cancer Discovery study by Pushalkar et al., entitled The Pancreatic Cancer Microbiome Promotes Oncogenesis by Induction of Innate and Adaptive Immune Suppression. This study suggested that bacteria within pancreatic tumors can promote tumor progression by inducing both innate and adaptive immune suppression, thereby providing important evidence linking intratumoral microbiota to cancer immunity. Another strong burst reference was the 2019 *Cell* article by Riquelme et al., entitled Tumor Microbiome Diversity and Composition Modulate Pancreatic Cancer Survival. This study showed that the diversity and composition of the intratumoral microbiota in pancreatic cancer are closely associated with patient survival, further extending the link between intratumoral microbiota and clinical outcomes.

Notably, aside from these relatively few studies directly focused on intratumoral microbiota, most of the remaining burst references still centered on gut microbiota and responses to immunotherapy. This finding suggests that although research on intratumoral microbiota in cancer immunotherapy has begun to establish a preliminary core literature base, its knowledge system still relies heavily on the broader conceptual framework of gut microbiota and immunotherapy research.

### 3.7 Keyword analysis

Keyword analysis showed that research hotspots in intratumoral microbiota and cancer immunotherapy have gradually evolved from early studies of immune mechanisms toward microbial biomarker development and microbiota-targeted intervention strategies. As shown in **Table 6**, “intratumoral microbiota” was the most frequently occurring keyword (N = 90), indicating its central position in the field’s research framework. In addition, keywords such as “tumor microenvironment,” “gut microbiota,” “intratumoral bacteria,” “colorectal cancer,” and “cancer therapy” each appeared more than 15 times, suggesting that current research mainly focuses on intratumoral microbial ecology, the host immune microenvironment, and their associations with specific tumor types and therapeutic strategies.

**Table 6:** Top 20 most frequent keywords.

Rank	Keywords	Count	Centrality	Year	Rank	Keywords	Count	Centrality	Year
1	intratumoral microbiota	90	0.83	2019	11	fecal microbiota transplantation	9	0.23	2020
2	tumor microenvironment	40	0.37	2020	12	lung cancer	6	0.02	2023
3	gut microbiota	32	0.65	2019	13	genetically engineered bacteria	5	0.40	2022
4	intratumoral bacteria	15	0.51	2022	14	gastric cancer	5	0.24	2023
5	colorectal cancer	15	0.40	2020	15	microbial metabolites	5	0.10	2022
6	cancer therapy	15	0.31	2017	16	bacterial therapy	4	0.16	2022
7	breast cancer	13	0.07	2021	17	immune system	4	0.06	2022
8	tumor immune microenvironment	11	0.37	2024	18	immune microenvironment	4	0.02	2022
9	cancer immunotherapy	11	0.24	2021	19	hepatocellular carcinoma	4	0.00	2025
10	immune checkpoint inhibitors	10	0.22	2019	20	immune modulation	4	0.00	2024

**Figure 8:**
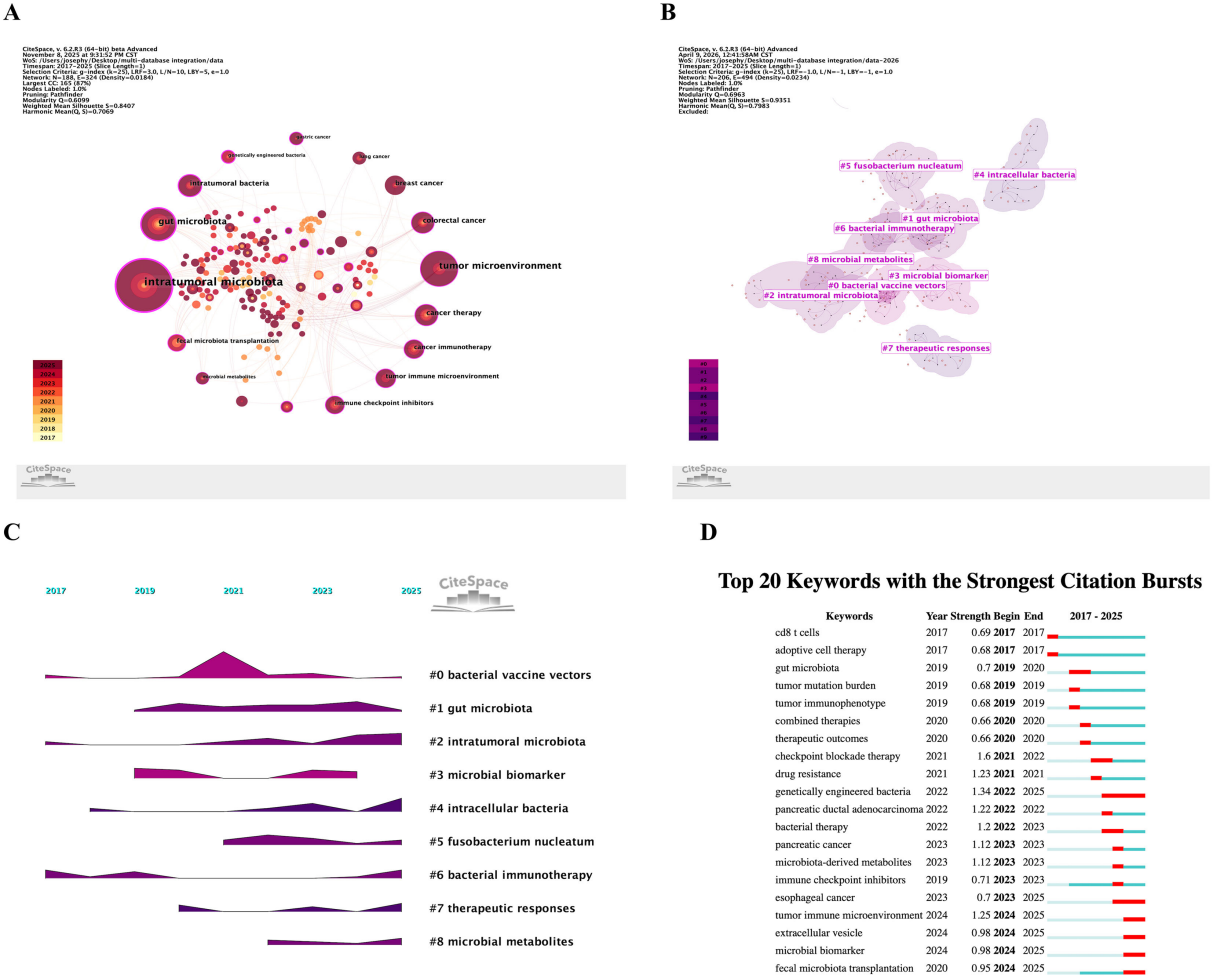
Keyword-based mapping of research landscape and hotspot evolution. **(A)** Keyword co-occurrence network of keywords with at least five occurrences. Node size is proportional to keyword frequency. **(B)**Keyword clustering analysis. Nine major clusters (Clusters #0-8) are identified. Clustering quality metrics: Modularity Q = 0.6963, Silhouette S = 0.9351. **(C)** Timeline visualization of keyword clusters from 2017 to 2025. **(D)** Keyword citation burst analysis of the top 20 keywords with the strongest citation bursts. Red bars indicate burst periods; blue line represents the timeline (2017-2025).

**Figure 8A** shows the keyword co-occurrence network based on keywords with at least five occurrences. The results indicated that “intratumoral microbiota,” “gut microbiota,” and “tumor microenvironment” formed the central high-frequency nodes in the network. Meanwhile, keywords such as “colorectal cancer,” “breast cancer,” “lung cancer,” and “gastric cancer” suggested that the research scope had expanded to multiple solid tumor types. In addition, terms such as “microbial metabolites,” “immune checkpoint inhibitors,” and “tumor immune microenvironment” indicated a gradual shift in research focus from descriptive observations to immune regulatory mechanisms and therapeutic response analysis.

Based on the keyword co-occurrence analysis, clustering analysis further identified nine major research clusters (**Figure 8B**). The clustering quality was high, with a modularity Q of 0.6963 and a silhouette S of 0.9351, indicating a clear clustering structure and high internal consistency. The identified clusters were #0 “bacterial vaccine vectors,” #1 “gut microbiota,” #2 “intratumoral microbiota,” #3 “microbial biomarker,” #4 “intracellular bacteria,” #5 “fusobacterium nucleatum,” #6 “bacterial immunotherapy,” #7 “therapeutic responses,” and #8 “microbial metabolites.” From the network structure, #2 “intratumoral microbiota” and #1 “gut microbiota” were located in the core area of the network and together formed the basic research framework of the field. Clusters #3 “microbial biomarker” and #7 “therapeutic responses” pointed to research directions involving treatment response prediction and clinical evaluation. Clusters #0 “bacterial vaccine vectors” and #6 “bacterial immunotherapy” reflected the emerging use of microorganisms as intervention tools. Cluster #8 “microbial metabolites” indicated that functional products involved in immune regulation are becoming an important emerging topic. In addition, #4 “intracellular bacteria” and #5 “fusobacterium nucleatum” formed relatively independent subclusters, suggesting that specific microbial taxa and their intratumoral localization patterns have developed into distinct research branches.

The timeline view (**Figure 8C**) showed marked differences in the onset and persistence of activity across research directions. Among them, #1 “gut microbiota” and #4 “intracellular bacteria” were the earliest emerging clusters, both beginning around 2017; #1 remained active throughout the study period, whereas #4 showed renewed activity in later years. Cluster #2 “intratumoral microbiota” emerged mainly around 2019 and remained active in the later period. Cluster #3 “microbial biomarker” was particularly active around 2019–2020, whereas #5 “fusobacterium nucleatum” and #7 “therapeutic responses” became increasingly prominent after 2020. Meanwhile, #8 “microbial metabolites” mainly emerged after 2022.

Keyword burst analysis (**Figure 8D**) further divided the evolution of this field into three consecutive stages. In the first stage (2017–2018), the focus was primarily on immune mechanism, as reflected by burst keywords such as “CD8^+^ T cells” (burst strength = 0.69), “adoptive cell therapy” (burst strength = 0.68), and “gut microbiota” (burst strength = 0.70), indicating that research mainly centered on effector T cell-mediated antitumor immunity and microbe–host interactions. In the second stage (2019–2021), the research focus gradually shifted toward molecular characterization and therapeutic optimization, with burst keywords such as “tumor mutation burden” (burst strength = 0.68), “tumor immunophenotype” (burst strength = 0.68), and “combined therapies” (burst strength = 0.66). At the same time, the emergence of “checkpoint blockade therapy” (burst strength = 1.60) and “drug resistance” (burst strength = 1.23) indicated that optimization of ICI efficacy and investigation of resistance mechanisms had become central topics during this phase. In the third stage (2022–2025), the field showed a clear translational orientation. Intervention-related keywords such as “genetically engineered bacteria” (burst strength = 1.34), “extracellular vesicle” (burst strength = 0.98), and “fecal microbiota transplantation” (burst strength = 0.95) continued to emerge, suggesting that microbiota-targeted strategies, including engineered bacteria, bacteria-derived extracellular vesicles (BEVs), and fecal microbiota transplantation have become new research frontiers. Meanwhile, bursts in “microbiota-derived metabolites” (burst strength = 1.12) and “microbial biomarker” (burst strength = 0.98) further indicated growing interest in the potential application of microbial metabolites and biomarkers in precision diagnosis and treatment.

Taken together, the results of keyword co-occurrence, clustering, timeline, and burst analyses consistently indicate that the field has moved beyond its early stage of mechanistic exploration and is entering a new phase oriented toward therapeutic response prediction and precision intervention.

## 4 Discussion

### 4.1 Overall research development trends

Research on intratumoral microbiota in cancer immunotherapy has grown rapidly in recent years. Since the first relevant publication appeared in 2017, annual publication output has increased steadily, with a marked acceleration after 2021. Between 2017 and 2024, publication output followed a significant exponential growth trajectory (CAGR = 63.1%, *p* < 0.0001), indicating that the field has moved rapidly from an initial exploratory phase into accelerated expansion and remains in an active growth stage rather than having reached a stable plateau.

One major driver of this rapid growth is the evolving landscape of cancer immunotherapy. ICIs have substantially improved clinical outcomes across multiple solid tumors. However, overall response rates remain limited, and both primary and acquired resistance are frequently observed (3, 12). In this context, identifying novel components of the TME that may explain therapeutic heterogeneity, contribute to immunosuppressive niche formation, and influence treatment response has become a central focus in tumor immunology. Located within the tumor niche, intratumoral microbiota have been increasingly investigated as tumor-local factors that may connect immune microenvironmental features with heterogeneous immunotherapy outcomes.

Technological advances have provided essential methodological support for this field. The development of single-cell sequencing, spatial transcriptomics, and high-sensitivity microbial detection techniques has enabled the identification of microbial signals in low-biomass tumor tissues and facilitated characterization of their spatial relationships with host cells, immune infiltration patterns, and local tissue architecture (13, 14). These advances have progressively challenged the long-standing assumption that tumor tissues are sterile and have driven the field from early detection and descriptive analyses toward functional and mechanistic investigations.

Importantly, research on intratumoral microbiota did not emerge in isolation, but rather developed as an extension and refinement of the broader framework linking microbiota, immunity, and tumor biology. Between 2017 and 2019, studies on gut microbiota and immunotherapy response established the conceptual foundation that microbial communities can modulate antitumor immunity and influence therapeutic outcomes. For example, landmark studies by Gopalakrishnan et al. and Routy et al. demonstrated strong associations between gut microbiota composition and responses to anti–PD-1 therapy (15, 16), providing systemic evidence for microbiota-mediated regulation of immunotherapy efficacy. Building on this foundation, research attention subsequently shifted from the gut to the tumor-local microenvironment. In 2020, Nejman et al. systematically characterized tumor type–specific microbial communities across multiple cancers (1), marking a key transition toward a more clearly defined and relatively independent line of research centered on intratumoral microbiota.

From an overall developmental perspective, the field has progressed from broad microbiota-associated immune regulation to detailed analyses of intratumoral microbial mechanisms, and more recently toward therapeutic response prediction and microbiota-targeted intervention. Early studies focused primarily on gut microbiota, CD8^+^ T-cell–mediated immunity, and fundamental immune mechanisms. This focus has gradually shifted toward immunotherapy resistance, microbial biomarkers, and intervention strategies such as engineered bacteria, extracellular vesicles (EVs), and fecal microbiota transplantation. This evolutionary pattern indicates that research on intratumoral microbiota in cancer immunotherapy is no longer confined to descriptive or mechanistic exploration, but is increasingly emerging as an interdisciplinary field that integrates mechanistic depth with translational potential.

### 4.2 Global research landscape and collaboration patterns

The global research landscape of intratumoral microbiota in cancer immunotherapy is characterized by a pattern in which China leads in publication output, while the United States demonstrates greater academic influence and plays a more central role in international collaboration networks. Although China contributes the largest number of publications, the United States surpasses other countries in total citations, network connectivity, and international academic reach. This pattern suggests that, in emerging interdisciplinary fields, research productivity and scientific influence do not necessarily evolve in parallel, but are shaped by multiple factors, including the timing of entry into the field, the international visibility of leading research groups, position within collaboration networks, and the impact of representative studies.

A notable feature of this field is the incomplete overlap between high productivity and high impact. The rapid increase in publications from China reflects sustained investment in microbiome research, tumor immunology, and translational medicine, as well as the ability of multiple research groups to rapidly engage with emerging hotspots. In contrast, the United States continues to occupy a more central position in terms of citation impact and international collaboration, indicating stronger influence in knowledge dissemination and academic recognition. Such disparities are not uncommon in rapidly expanding research areas, where some countries achieve large-scale output in a short period, whereas foundational knowledge systems and long-term academic influence are often shaped by research centers that entered the field earlier, consistently produced high-impact studies, and are deeply embedded in global collaboration networks.

The structure of international collaboration further highlights the United States as a key hub in global knowledge exchange. The United States maintains the strongest bilateral collaboration with China while also forming extensive partnerships with countries such as Italy, the Netherlands, and the United Kingdom. In contrast, despite its high publication volume, China shows a relatively low proportion of multinational collaborations, suggesting that its research output is still largely driven by domestic research groups. These differences reflect distinct patterns of research organization. The United States tends to rely on open and interconnected international collaboration networks to integrate resources, disseminate methodologies, and enhance global visibility, whereas China’s contribution is more strongly characterized by the rapid expansion of densely connected domestic research clusters. For a field such as intratumoral microbiota research, which requires high-quality samples, sensitive detection in low-biomass settings, advanced spatial analysis, and strong interdisciplinary integration, collaboration network centrality affects not only the dissemination of findings but also study design rigor, methodological standardization, and the ability to validate results across independent cohorts.

At the same time, several European countries, although contributing fewer publications overall, show relatively high citation impact per article and strong participation in international collaborations. This pattern illustrates that a smaller-scale but highly connected research model can also generate substantial academic influence. It also indicates that, in emerging interdisciplinary fields, scientific impact is not solely determined by publication volume. High-quality research addressing key scientific questions, integration into high-level international networks, and contributions to foundational discoveries or methodological innovation are equally important for enhancing academic visibility and citation impact. As a result, the global research landscape is not defined simply by publication volume, but rather reflects differences among countries in productivity, collaboration strategies, and forms of knowledge contribution.

This country-level pattern is further supported by institutional- and author-level analyses. China has developed several major research clusters centered on institutions such as Nanjing Medical University, the Chinese Academy of Sciences, Zhejiang University, and Sun Yat-sen University. In contrast, The University of Texas MD Anderson Cancer Center remains highly prominent in terms of publication output, citation impact, and international connectivity. At the author level, collaboration networks sustained relatively fragmented, with cross-national collaborations often maintained by a limited number of highly influential researchers. These observations suggest that, although a global research network has begun to take shape, it remains insufficiently integrated and has not yet evolved into a mature system characterized by stable, well-structured, and highly coordinated collaboration.

From a temporal perspective, the field appears to be transitioning from an early stage dominated by European and American institutions to a more multipolar structure, with China emerging as a major contributor in publication output, the United States serving as a central collaboration hub, and European countries participating as interconnected nodes. This shift reflects both the rapid rise of research capacity in China and the broader expansion of the field from a limited number of pioneering groups to a globally distributed research community. Moving forward, progress in this field will depend on strengthening cross-national, cross-institutional, and interdisciplinary collaborations, particularly in areas such as sample standardization, spatially resolved analyses, mechanistic validation, and clinical translation. Such efforts will be essential for translating high publication output into sustained and internationally recognized scientific impact.

### 4.3 Knowledge base and disciplinary structure

The co-citation network, dual-map overlay, and burst reference analysis collectively indicate that research on intratumoral microbiota in cancer immunotherapy is grounded primarily in immunology and microbiology, rather than representing a simple extension of conventional oncology research. High-impact multidisciplinary journals, including Science, Nature, Cell, and Nature Medicine, constituted the major sources of co-cited knowledge, suggesting that the conceptual foundation of this field has been shaped mainly by basic studies of tumor immunity, host-microbe interactions, and tumor-local microbial ecology.

A notable feature of the disciplinary structure is the distinction between publication venues and co-cited knowledge sources. In recent years, studies in this field have been published mainly in immunology-, oncology-, and translational medicine-oriented journals, such as Frontiers in Immunology, Cancers, Frontiers in Oncology, and Journal of Translational Medicine. By contrast, the most frequently co-cited references remain concentrated in high-impact basic research journals, including Science, Nature, Cell, and Nature Medicine. This contrast suggests that, although current research output is increasingly hosted by specialized journals with clearer disciplinary positioning, the theoretical basis of the field still relies heavily on foundational discoveries published in high-impact general science and biomedical journals.

More importantly, the co-citation network, highly cited references, and burst references reveal several key conceptual transitions during the formation of this field. These representative studies not only constitute the knowledge base of intratumoral microbiota research, but also reflect the progressive expansion of scientific questions from systemic microbiota-immune interactions to tumor-local microbial ecology and translational applications.

The first conceptual transition was from gut microbiota and systemic immune regulation to a broader microbiota-immunity-cancer framework. Burst references represented by Routy et al. (16), Gopalakrishnan et al. (15), and Matson et al. (17) demonstrated that gut microbiota composition is associated with responses to anti-PD-1 therapy. These studies challenged the earlier view that immunotherapy efficacy is determined mainly by tumor-intrinsic and host genetic factors, and established microbial communities as important host-associated variables linked to antitumor immune responses. By linking gut microbiota to systemic variation in ICI response, these studies provided the conceptual basis for subsequent investigations of microbiome-related mechanisms in cancer immunotherapy.

The second conceptual transition was from systemic microbiota-mediated immune modulation to tumor-local microbial ecology. Studies by Pushalkar et al. (18) and Riquelme et al. (19) extended the research focus from the gut to the tumor tissue itself by showing that microbial communities within pancreatic tumors are associated with local immune regulation, tumor progression, and patient prognosis. In this context, the study by Nejman et al. (1) represented a landmark advance in the field. By systematically characterizing tumor type-specific intracellular bacteria across multiple human cancers, this work provided important evidence for the presence of microbial communities within tumor tissues and stimulated broad interest in the tumor-local microbial ecosystem. Compared with earlier studies that mainly emphasized gut microbiota-mediated regulation of host immunity, this line of research shifted attention toward the composition, spatial distribution, and potential biological functions of intratumoral microbiota.

The third conceptual transition was from descriptive detection of intratumoral microbes to functional validation and translational relevance. Subsequent studies represented by Fu et al. (20), Kalaora et al. (21), and Galeano Niño et al. (5) further advanced the field by investigating the potential functions of intratumoral microbiota in tumor metastasis, antigen presentation, immune regulation, and the spatial organization of the TME. These studies suggest that the field is moving beyond descriptive ecological observations and toward mechanistic interrogation of how tumor-associated microbial signals may relate to tumor immunity and therapeutic responses. Accordingly, intratumoral microbiota have increasingly been investigated as a tumor-local factor potentially linked to the TIME and heterogeneous responses to cancer immunotherapy.

Taken together, co-citation and burst reference analyses suggest that the theoretical development of this field can be summarized in three major advances. First, microbiota research broadened the conceptual framework of cancer immunotherapy. It highlighted microbial communities as host-associated factors that may be linked to antitumor immune responses. Second, the identification of microbial communities within tumor tissues provided a new perspective for understanding tumor-local ecological niches. Third, the field has gradually evolved from evidence of microbial presence toward functional studies addressing immune regulation, tumor progression, and therapeutic response. These key studies collectively form the current knowledge base of intratumoral microbiota research in cancer immunotherapy, continue to shape the major scientific questions in the field, and provide an important foundation for future mechanistic studies and clinical translation.

### 4.4 Research hotspots: from microbial mechanisms to biomarkers and interventions

Keyword co-occurrence, clustering, timeline visualization, and burst analyses indicate that research hotspots in intratumoral microbiota and cancer immunotherapy have evolved from broad association-based observations toward three interrelated directions. The first concerns the transition from gut microbiota-centered frameworks to tumor-local microbial ecology. The second concerns the use of intratumoral microbial features as potential biomarkers for ICI response and resistance. The third concerns the development of microbiota-targeted interventions, including engineered bacteria, microbial-derived effectors, and ecosystem-level modulation. Together, these directions reflect a logical progression from theoretical framing to biomarker development and, ultimately, therapeutic translation.

#### 4.4.1 From gut microbiota-centered frameworks to tumor-local microbial ecology

A central feature of hotspot evolution in this field is the shift from an early gut microbiota-centered framework to a more focused interest in tumor-local microbial ecology. In the early stage, burst keywords such as “gut microbiota” and “CD8^+^ T cells” suggested that researchers were primarily concerned with how microbial communities influence systemic antitumor immunity and immunotherapy outcomes. These studies established a broad microbiota-immunity-cancer framework, showing that microbial communities are not passive bystanders in cancer therapy but host-associated factors linked to immune responsiveness and clinical outcomes. This conceptual foundation provided the starting point for extending microbiome research from the gut to the tumor-local microenvironment.

However, a gut-centered framework alone cannot fully explain how microbial signals operate across anatomically distinct host compartments. The concept of microbiome compartmentalization has therefore become useful for interpreting the divergence between gut microbiota and intratumoral microbiota. Gut microbiota primarily affect tumors through systemic host-mediated pathways (22, 23), including microbial metabolites and other gut-derived signals (24), inflammatory and cytokine signaling (25), peripheral T-cell differentiation (26), and broader host immune education (15). In contrast, intratumoral microbiota are embedded within tumor tissues, tumor cells, stromal compartments, or immune-rich niches. Their close spatial proximity to tumor and immune cells makes them particularly relevant to localized immune regulation, tumor type-specific ecological states, and regional heterogeneity in treatment response (22, 23, 27).

Against this background, research attention has increasingly converged on tumor-local mechanisms. The key questions have shifted from whether microbes are associated with immunotherapy outcomes to which microbes are present within tumors, where they are located, and how their spatial distribution may relate to TIME remodeling and ICI responsiveness. Current studies have highlighted several possible local mechanisms, including changes in myeloid cell composition, immune checkpoint-related signaling, effector T-cell infiltration and function, and microbial metabolite or antigen production within the tumor niche (28–30). For example, studies in pancreatic cancer have linked intratumoral bacteria to innate and adaptive immune suppression (18), whereas work on Fusobacterium nucleatum in colorectal cancer has connected specific bacterial signals with impaired antitumor immunity (31).

Another important trend is the move from compositional profiling to functional and spatial analysis. Recent studies have begun to examine microbial metabolites, bacterial antigens, EVs, and intracellular versus extracellular localization as biologically meaningful features of the tumor microbiome (32–34). For instance, microbial metabolites have been implicated in the regulation of PD-L1 expression, chemokine signaling, and CD8^+^ T-cell recruitment (35, 36). In melanoma, bacterially derived HLA-binding peptides have been shown to be presented to the immune system and to elicit antigen-specific T-cell responses (21). Moreover, emerging evidence suggests that the immune relevance of intratumoral bacteria may depend not only on bacterial identity but also on their intracellular or extracellular localization (37). These findings indicate that research hotspots are no longer limited to cataloguing microbial taxa. Instead, the field is moving toward spatially resolved and functionally oriented questions centered on tumor-local host-microbe interactions.

Overall, this transition from gut microbiota-centered frameworks to tumor-local microbial ecology marks the maturation of intratumoral microbiota research. Gut microbiota studies provided the initial conceptual entry point, but the field is now developing a more specific set of questions around microbial spatial localization, localized immune regulation, and tumor type-specific host-microbe interactions. This evolution explains why keywords such as intratumoral microbiota, intracellular bacteria, microbial metabolites, and therapeutic responses have become increasingly prominent in recent years.

#### 4.4.2 From immune regulation to microbial biomarkers of ICI response

As the potential role of intratumoral microbiota in shaping the TIME has received increasing attention, research hotspots have shifted toward their relevance to ICI response, resistance, and patient stratification. This transition reflects a change in research objectives. The field is moving from asking whether intratumoral microbes participate in local immune regulation to exploring whether microbial features can help explain interpatient variability in immunotherapy outcomes. Consistent with this shift, terms related to drug resistance, checkpoint blockade therapy, therapeutic responses, and microbial biomarkers have become more prominent in keyword and burst analyses.

This shift has a clear clinical rationale. Although ICIs have improved outcomes in multiple solid tumors, only a subset of patients achieve durable benefit. Conventional predictive markers, such as PD-L1 expression, tumor mutational burden, and immune phenotypes, provide useful but incomplete information (38). Intratumoral microbiota may provide an additional layer of tumor-local biological information. Their spatial positioning within the tumor niche enables investigation of their associations with immune cell infiltration, immune checkpoint-related signaling, inflammatory states, and metabolic conditions. Therefore, microbiota-related features are increasingly being investigated as potential contributors to treatment heterogeneity rather than as stand-alone determinants of ICI efficacy (39, 40).

Current evidence suggests that intratumoral microbiota may be linked to immune states associated with ICI responsiveness. Spatial transcriptomic and single-cell studies in tumors such as oral squamous cell carcinoma and colorectal cancer have reported that microbe-enriched regions can be associated with reduced vascularization, immunosuppressive features, myeloid cell infiltration, immune checkpoint upregulation, and decreased T-cell marker expression (5). In head and neck squamous cell carcinoma, higher intratumoral bacterial burden has been associated with reduced CD8^+^ T-cell infiltration, expansion of immunosuppressive cell populations, and poorer response to PD-1/PD-L1-targeted therapies (41). These observations support the possibility that microbiota-associated resistance may reflect broader remodeling of the local immune ecosystem rather than a single molecular event.

At the same time, the association between intratumoral microbiota and ICI response is not uniformly suppressive and may vary according to microbial species, tumor type, spatial localization, and the local immune milieu. Some microbial species have been associated with enhanced immune activation and improved response to anti-PD-1 therapy. For example, RNA-sequencing-based analyses of ICI-treated cohorts identified several bacterial species associated with improved anti-PD-1 response. Experimental models further suggested that local administration of selected bacteria could enhance antitumor immune responses when combined with anti-PD-1 therapy (6). Such findings indicate that intratumoral microbes may include both unfavorable and potentially beneficial signals, depending on microbial species, tumor type, spatial localization, and local or systemic immune conditions.

Beyond immune cell infiltration and function, intratumoral microbiota may also be associated with tumor cell-intrinsic pathways relevant to ICI responsiveness. In colorectal cancer, enrichment of enterotoxigenic *Bacteroides fragilis* after capecitabine and PD-1 blockade has been linked to increased sphingosine-1-phosphate production, altered chromatin accessibility at the *CD274* promoter, and enhanced PD-L1 expression. These changes were accompanied by expansion of effector memory CD8^+^ T cells and a reduction in exhausted T-cell populations (42). Together, these observations suggest that intratumoral microbial signals may be associated not only with immune microenvironmental changes but also with tumor cell states that influence immune checkpoint dependence.

Building on these observations, microbial biomarkers have become a major emerging direction. Compared with conventional host- or tumor-derived markers, microbiota-related features may provide information about spatial specificity, ecological dynamics, and host-microbe interactions within the TME. Future biomarker studies should therefore move beyond the presence or absence of individual taxa and examine integrated signatures that include microbial species, metabolites, spatial distribution patterns, local immune features, and treatment regimens. Such features are more likely to complement existing predictive models than to replace established biomarkers.

Before intratumoral microbiota can be used as reliable predictive biomarkers, their reproducibility across cohorts, tumor types, and analytical platforms must be established (43). At present, most microbiota-related features remain exploratory and should be viewed as complementary to existing predictive markers rather than as stand-alone indicators of ICI efficacy. Their clinical value will require standardized detection procedures, independent validation, and prospective evaluation in well-defined patient cohorts, together with host immune, molecular, and clinical parameters.

Overall, this hotspot shift extends the scope of intratumoral microbiota research from local immune regulation to clinically relevant applications in treatment prediction and patient stratification. However, bibliometric and association-based evidence should be interpreted cautiously. Intratumoral microbiota are best viewed at present as emerging candidate biomarkers and biologically relevant tumor-local factors whose clinical utility requires further mechanistic and prospective validation.

#### 4.4.3 From association-based findings to microbiota-targeted interventions

As evidence connecting intratumoral microbiota with tumor immunity and immunotherapy outcomes has accumulated, research has increasingly moved from association-based observations toward microbiota-targeted interventions. Early studies mainly described the presence, composition, and heterogeneity of intratumoral microbes across cancer types and examined their associations with immune phenotypes and clinical outcomes. More recent work has begun to ask whether microbial communities or their functional outputs can be manipulated to enhance antitumor immunity and improve ICI responsiveness. This transition indicates that the field is moving from descriptive mapping toward translational exploration.

This shift is driven by both mechanistic rationale and clinical need. Accumulating links between intratumoral microbial signals, local immune states, and treatment outcomes have raised interest in microbiota-based combination strategies. Compared with conventional host-directed strategies, microbiota-based approaches may act within the TME by exploiting microbial metabolic activity, immune-stimulatory potential, and delivery capacity. Accordingly, increasing attention has focused on engineered bacteria, naturally beneficial strains, BEVs, microbial metabolites, bacterial antigens, and broader microbiota-remodeling strategies.

One major intervention strategy involves engineered or naturally beneficial bacteria. Some bacteria exhibit tumor tropism and can be genetically engineered to deliver therapeutic agents, immune modulators, or other functional cargo within the TME (8, 44). In parallel, bacterial species associated with improved ICI response are being explored as potential local immune modulators (6). These efforts reflect a shift from identifying tumor-associated microbes to determining whether selected organisms can be used as therapeutic vehicles or immune-regulatory tools. Nevertheless, the clinical development of live bacterial or engineered bacterial therapies requires rigorous evaluation of safety, stability, colonization control, dose regulation, and off-target effects.

A second strategy targets microbial-derived effectors rather than intact microbes. As the field has evolved, therapeutic interest has expanded to metabolites, bacterial antigens, and EVs. These approaches may offer improved controllability and standardization compared with live bacterial administration and may be easier to combine with existing immunotherapy regimens. For example, BEVs and specific microbial metabolites have been reported to modulate immune cell function, immune checkpoint-related signaling, or antigen presentation (45). This direction suggests that therapeutic development may not need to focus solely on changing microbial composition, but can also target microbial functions and downstream immune consequences.

A third strategy involves ecosystem-level modulation, with fecal microbiota transplantation (FMT) being the best-known example. Although FMT originated in gut microbiota research, its ability to remodel systemic microbial ecosystems and influence immunotherapy response has brought it into discussions of tumor-associated microbial regulation (17, 19, 46). From the perspective of intratumoral microbiota research, FMT and related approaches may indirectly affect tumor microbial colonization, microbial metabolites, systemic immune conditioning, and local tumor immunity. This does not mean that FMT directly targets intratumoral microbiota in all settings, but it highlights the need to consider coordinated interactions between systemic and tumor-local microbial compartments.

The transition from association to intervention also changes the standards for evaluating progress. Descriptive studies have primarily focused on the presence of intratumoral microbes and their correlations with immune phenotypes or clinical outcomes. Intervention-oriented studies must instead define which microbial species, functional mediators, delivery systems, and combination regimens can produce reproducible therapeutic effects with acceptable safety. This requires integration of microbial biology, spatial localization, functional validation, tumor immune features, and clinically relevant endpoints into testable therapeutic designs.

Despite increasing interest, several barriers remain before microbiota-targeted interventions can be translated into routine clinical practice. Intratumoral microbiota vary across tumor types, anatomical sites, spatial niches, and host immune or metabolic conditions. Effective strategies may therefore need to be tailored to specific cancer types, microbial configurations, immune states, and treatment settings. In addition, most evidence remains preclinical or exploratory. It is also unclear how microbiota-targeted strategies should be combined with ICIs, chemotherapy, radiotherapy, targeted therapy, or other immunomodulatory approaches. Standardized detection, contamination control, mechanism-guided selection, and prospective clinical validation will be essential for future development.

Overall, microbiota-targeted intervention represents an important emerging frontier in intratumoral microbiota research. The field is no longer limited to describing microbial presence or explaining immune associations. It is increasingly oriented toward functional validation and translational exploration. However, rather than viewing intratumoral microbiota as fully programmable components of precision cancer therapy at this stage, it may be more appropriate to regard them as promising tumor-local biological targets and intervention-related resources whose therapeutic potential remains to be validated through standardized mechanistic studies and clinical trials.

### 4.5 Methodological challenges and spatial multi-omics validation

Despite the rapid growth of intratumoral microbiota research, the field remains constrained by several methodological challenges. The most fundamental challenge is the low-biomass nature of tumor-associated microbial signals. In their analysis of more than 1,500 tumor samples across seven cancer types, Nejman et al. showed that microbial signals in solid tumors are generally low in abundance and vary markedly across tumor types. In some tumor types, detectable bacterial DNA signals were present only in a small fraction of samples, approaching the lower limit of reliable detection by conventional sequencing platforms (1). Under such conditions, even trace amounts of exogenous DNA can substantially influence microbial profiles and amplify uncertainty in data interpretation.

Contamination represents a major source of systematic bias in low-biomass microbiome studies. Reanalysis of TCGA whole-genome sequencing data by Ge et al. suggested that some previously reported tumor microbial signals may have been overestimated because of contamination and sequence misassignment (47). Salter et al. further demonstrated that commercial DNA extraction kits and PCR reagents can contain stable background microbial DNA (48), while van der Valk et al. showed that index hopping during sequencing can generate false-positive microbial signals, particularly in low-biomass samples (49). These observations highlight the need for rigorous contamination control. Negative controls, extraction blanks, reagent controls, mock communities, standardized reporting procedures, and computational decontamination strategies should be considered essential components of intratumoral microbiota research. In this regard, Eisenhofer et al. emphasized that strict negative-control systems and standardized contamination-control workflows, such as the RIDE framework, are essential prerequisites for low-biomass microbiome studies (50).

In addition to contamination, the lack of standardization across experimental and analytical workflows further limits comparability among studies. DNA extraction protocols can introduce taxon-specific biases in bacterial recovery, as shown in multicenter experimental analyses of microbiome sample processing (51). Differences in 16S rRNA hypervariable region selection, PCR conditions, sequencing depth, sequencing platforms, batch effects, and bioinformatic pipelines can also influence inferred microbial composition (52). These technical differences make it difficult to compare results across cohorts and studies, and they may also affect downstream analyses of microbial biomarkers, co-occurrence patterns, and associations with immunotherapy outcomes. Therefore, standardized sample processing, sequencing procedures, quality-control criteria, and analytical pipelines are necessary for improving reproducibility in this field.

Spatial validation represents another critical methodological requirement. Bulk sequencing can identify microbial reads in tumor specimens, but it cannot determine whether these signals originate from tumor cells, stromal compartments, immune-rich niches, adjacent tissues, necrotic regions, or contamination. This limitation restricts the biological interpretation of intratumoral microbial signals and has prompted increasing interest in spatially resolved microbial detection and validation (53). Spatially resolved approaches, including fluorescence *in situ* hybridization, RNAscope-based microbial detection, spatial transcriptomics, multiplex imaging, and related imaging-based methods, provide important tools for verifying the localization of microbial signals within tumor tissues. These approaches are particularly important for determining whether microbial signals are located within tumor cells, stromal compartments, immune-rich niches, or extracellular regions. They also provide a basis for distinguishing true tumor-local signals from contamination, necrotic-region artifacts, or signals derived from adjacent tissues. More broadly, spatial transcriptomics enables simultaneous assessment of host transcriptional states and tissue architecture *in situ*, thereby improving the resolution of tumor spatial heterogeneity (54). For example, Galeano Niño et al. used spatially resolved analyses to map bacterial signals in oral squamous cell carcinoma and colorectal cancer, providing evidence that microbial signals can be spatially organized within tumor tissues and associated with distinct features of the TME (5).

The field is now moving from spatial localization toward spatial functional interpretation. By integrating spatial transcriptomics, single-cell transcriptomics, metagenomic or microbial read analysis, metabolomics, and immune profiling, spatial multi-omics approaches can help connect microbial distribution with local immune states, metabolic niches, and therapeutic response patterns (54, 55). In this framework, microbial sequencing or microbial read analysis provides the initial detection of microbial signals. FISH or RNAscope can then support *in situ* validation of microbial localization (5, 56). Single-cell omics, spatial transcriptomics, multiplex imaging, and metabolomics further connect microbial distribution with immune cell states, tissue architecture, microbe-cell proximity, and local metabolic niches (57). Such approaches are particularly valuable for distinguishing true tumor-local microbial signals from technical artifacts and for identifying how microbial signals are spatially related to immune cell infiltration, antigen presentation, immune checkpoint-related pathways, and T-cell dysfunction. More specifically, they can help determine whether microbial signals are located near CD8^+^ T-cell-infiltrated regions, myeloid-enriched niches, TAMs, Tregs, MDSCs, or PD-L1-high areas, thereby linking microbial localization to local immunosuppressive or immune-reactive states. For instance, spatially defined multi-omics analyses in colorectal cancer have begun to link specific intratumoral microbial patterns with altered metabolic programs and immune cell states. Chen et al. integrated single-cell RNA sequencing, spatial transcriptomics, and microbiome multi-omics data to construct a spatially defined multimodal framework in colorectal cancer. This analysis linked the spatial depletion of specific microbial taxa, such as *Streptococcus* and *Acetitomaculum*, to disrupted butyrate metabolism and CD8^+^ T-cell exhaustion. Mechanistically, these changes were associated with a butyrate–HDAC–PDCD1 acetylation axis (58). This example illustrates how spatial multi-omics may provide a route from microbial signal detection to mechanistic interpretation.

Together, these methodological issues indicate that intratumoral microbiota research must move beyond microbial signal detection alone. Greater emphasis should be placed on contamination-aware study design, standardized analytical workflows, spatial validation, and cross-cohort reproducibility. Addressing these challenges will be essential for transforming intratumoral microbiota signals into reliable mechanistic insights and clinically testable biomarkers or intervention targets.

### 4.6 Future perspectives: a phased framework for clinical translation

Although research on intratumoral microbiota has advanced from microbial detection toward immune regulation, biomarker discovery, and intervention development, its clinical translation remains at an early stage. Most available evidence still comes from retrospective analyses, exploratory cohorts, or preclinical models. Rigorously designed multicenter prospective studies are still needed to determine whether intratumoral microbiota-related features can be translated into stable clinical biomarkers or therapeutic strategies. In this context, future clinical translation should be guided by a phased framework in which discovery, technical validation, clinical validation, and intervention development are connected as sequential but mutually reinforcing steps.

For microbial biomarker development, the first priority is to move from exploratory associations toward prospectively testable predictive models. Existing studies have already provided examples of clinically relevant microbial features. For instance, the CIAO clinical trial (NCT03144778) reported that total intratumoral bacterial burden was associated with ICI response in patients with head and neck squamous cell carcinoma (41). Notably, this association was linked to overall bacterial burden rather than the composition of specific bacterial taxa, and it was further evaluated in independent cohorts. Similarly, a cohort study of 802 patients with nasopharyngeal carcinoma reported that higher intratumoral bacterial burden was associated with reduced T-cell infiltration and poorer prognosis (59). These findings suggest potential clinical value, but they should be regarded as starting points for prospective validation rather than definitive clinical tools.

The next step is to evaluate whether microbial features provide added value beyond established biomarkers. Candidate models should be developed in training cohorts, tested in internal validation sets, and further assessed in external independent cohorts. Multivariable modeling and decision curve analysis may help determine whether microbial features improve prediction beyond PD-L1 expression, tumor mutational burden, immune infiltration patterns, molecular subtypes, and standard clinicopathological variables. In this process, technical validation should build on the methodological standards discussed above and focus on whether candidate microbial features remain reproducible across centers, platforms, sampling procedures, and analytical pipelines.

For clinical validation, multicenter prospective cohorts should be designed with prespecified endpoints. Depending on the intended use of the microbial marker, these endpoints may include objective response rate (ORR), disease control rate (DCR), progression-free survival (PFS), overall survival (OS), duration of response (DoR), and immune-related adverse events (irAEs). Rather than replacing existing biomarkers, intratumoral microbiota-related features are more likely to function as components of integrated predictive models. Such models should combine microbial information with immune, molecular, pathological, and clinical parameters to support patient stratification and treatment selection.

Microbiota-targeted interventions require a related but distinct translational pathway. Current evidence is beginning to connect mechanistic rationale with early clinical testing. For example, Chen et al. analyzed multiple ICI-treated cohorts and identified intratumoral bacterial species associated with anti-PD-1 sensitivity using tumor transcriptomic data. Experimental models further suggested that selected intratumoral bacteria could enhance the antitumor effects of anti-PD-1 therapy (6). In addition, a phase Ib clinical trial (NCT03435952) has evaluated the safety and tolerability of engineered anaerobic Clostridium novyi-NT in combination with pembrolizumab (60). These examples indicate that microbiota-based strategies are beginning to enter early translational testing, but their clinical value still requires staged evaluation.

Phase I studies should prioritize safety, tolerability, dose-limiting toxicity, and dose selection. For interventions involving live or engineered bacteria, colonization control should also be assessed. Preliminary biological activity may be evaluated as an exploratory endpoint. Phase II studies should then assess antitumor activity and biomarker-defined subgroup responses using endpoints such as ORR, DoR, PFS, and immune modulation. Phase III randomized trials will be needed to determine whether microbiota-targeted strategies provide clinically meaningful benefit when combined with ICIs or other standard therapies. In these trials, PFS and OS should serve as major efficacy endpoints, whereas irAEs and other treatment-related adverse events should be carefully monitored as key safety outcomes.

Overall, clinical translation of intratumoral microbiota research will depend on a phased validation strategy rather than isolated exploratory findings. A clinically useful framework should connect biomarker discovery with technical reproducibility, prospective clinical validation, and staged intervention development. This approach may help move the field from association-based observations toward clinically testable microbial biomarkers and microbiota-based therapeutic strategies.

### 4.7 Limitations

This study has several limitations. First, the field of intratumoral microbiota in cancer immunotherapy is still at an early stage of development. A total of 245 publications were included up to the search date, and the first relevant study was published only in 2017. Although this volume is sufficient for basic bibliometric analysis, the evidence base remains relatively small compared with more mature research areas. This may affect the stability of clustering analyses and the sensitivity of burst detection, and some low-frequency but potentially important research directions may not have been fully captured.

Second, limitations remain in database coverage and citation completeness. Although WoSCC, Scopus, and PubMed were combined to improve retrieval coverage, structural differences among these databases could not be completely eliminated. Given the less complete exportable reference metadata available from PubMed compared with WoSCC and Scopus, co-citation and citation analyses were based primarily on WoSCC and Scopus records. This may have affected the completeness of the resulting knowledge map.

Third, the search strategy involved a necessary trade-off between specificity and comprehensiveness. To improve topical relevance, the search was restricted to titles, abstracts, and author keywords, and only English-language articles and reviews were included. As a result, studies that discussed intratumoral microbiota and cancer immunotherapy only in the full text may have been missed. The exclusion of non-English publications may also have led to some underrepresentation of research activity from certain countries or regions. To assess this potential bias, we performed an additional search of the CNKI database. Only a limited number of Chinese-language records were identified, including eight academic theses and two review articles. The theses did not meet the predefined inclusion criteria, while the two reviews accounted for less than 1% of the included dataset. This suggests that the language restriction is unlikely to have substantially altered the overall research landscape. Nevertheless, future bibliometric studies incorporating multilingual databases may provide a more comprehensive view of global research activity.

Fourth, citation-based indicators are inherently time-dependent. In a rapidly expanding field, recently published studies may not yet have accumulated sufficient citations, even if they have high scientific relevance. In addition, self-citations at the author, institutional, or journal level were not separately excluded. Given the relatively small and rapidly evolving nature of this field, self-citation may have influenced citation counts, co-citation link strength, network structure, and burst detection results. Therefore, citation-based indicators should be interpreted as measures of scholarly visibility and research activity rather than direct measures of independent scientific impact.

Fifth, differences in author names, institutional affiliations, and bibliographic metadata are recorded across databases may introduce bias. Although Python was used for deduplication, cleaning, and standardization of authors, institutions, and related fields, some discrepancies may remain due to abbreviated names, inconsistent spelling, or entity-merging errors. Therefore, the collaboration networks should be interpreted primarily as representations of overall structural patterns rather than exact depictions of all individual relationships.

Finally, heterogeneity in the primary studies may affect bibliometric interpretation. As discussed in Section 4.5, intratumoral microbiota research is strongly influenced by differences in low-biomass microbial detection, contamination control, sequencing platforms, analytical pipelines, and spatial validation. Such methodological heterogeneity may affect keyword clustering, hotspot interpretation, and the apparent strength of links between microbial features, immune regulation, and immunotherapy response.

Taken together, these limitations do not alter the main conclusions of this study regarding the overall research landscape, knowledge base, and evolution of research hotspots in this field. They do, however, indicate that future bibliometric studies could be strengthened by incorporating broader and multilingual data sources, updating the dataset as the field expands, and further improving metadata and methodological standardization.

## 5 Conclusion

This bibliometric and visualization analysis shows that research on intratumoral microbiota in cancer immunotherapy has rapidly developed into a distinct and expanding interdisciplinary field. Research hotspots have shifted from early mechanistic exploration toward microbial biomarker discovery, therapeutic response prediction, and microbiota-targeted intervention. However, clinical translation remains constrained by several challenges, including limited standardization of low-biomass microbial detection, insufficient contamination control, predominance of retrospective study designs, and a lack of multicenter prospective validation. Future progress will require standardized analytical workflows, spatial and multi-omics validation, and well-designed prospective clinical studies. These efforts may help improve the reliability and reproducibility of intratumoral microbiota research and support the development of clinically testable microbial biomarkers and microbiota-based therapeutic strategies.
