# Supplementary material for: Global research trends and emerging frontiers of intratumoral microbiota in cancer immunotherapy: a bibliometric and visualization analysis
Source: Front Immunol. 2026 Jul 21;17:1865305. doi: 10.3389/fimmu.2026.1865305 (PMC13433859; doi:10.3389/fimmu.2026.1865305)
Supplement: Supplementary file 1 [file Table1.docx]

Document Type = (Article OR Review)

Language = English

limited to the Title, Abstract, and Author Keywords fields, excluding Keywords Plus

**WoSCC search strategies**

((TI=("intratumor* microbio*" OR "intratumor* bacter*" OR "tumor microbio*" OR "tumor bacter*" OR "tumor-resident microbio*" OR "tumor-resident bacter*" OR "tumor-infiltrating bacter*" OR "tumor-infiltrating microbio*")) OR (AK=("intratumor* microbio*" OR "intratumor* bacter*" OR "tumor microbio*" OR "tumor bacter*" OR "tumor-resident microbio*" OR "tumor-resident bacter*" OR "tumor-infiltrating bacter*" OR "tumor-infiltrating microbio*")) OR (AB=("intratumor* microbio*" OR "intratumor* bacter*" OR "tumor microbio*" OR "tumor bacter*" OR "tumor-resident microbio*" OR "tumor-resident bacter*" OR "tumor-infiltrating bacter*" OR "tumor-infiltrating microbio*"))) AND ((TI=("immunotherap*" OR "adoptive cell therap*" OR "CAR-T" OR "immune checkpoint blockade*" OR "immune checkpoint inhibitor*" OR "immune modulation" OR "ICI*" OR "CPI" OR "CTLA-4" OR "PD-1" OR "PD-L1" OR "Nivolumab" OR "Atezolizumab" OR "Sintilimab" OR "Pembrolizumab" OR "Ipilimumab" OR "Durvalumab" OR "Avelumab")) OR (AK=("immunotherap*" OR "adoptive cell therap*" OR "CAR-T" OR "immune checkpoint blockade*" OR "immune checkpoint inhibitor*" OR "immune modulation" OR "ICI*" OR "CPI" OR "CTLA-4" OR "PD-1" OR "PD-L1" OR "Nivolumab" OR "Atezolizumab" OR "Sintilimab" OR "Pembrolizumab" OR "Ipilimumab" OR "Durvalumab" OR "Avelumab")) OR (AB=("immunotherap*" OR "adoptive cell therap*" OR "CAR-T" OR "immune checkpoint blockade*" OR "immune checkpoint inhibitor*" OR "immune modulation" OR "ICI*" OR "CPI" OR "CTLA-4" OR "PD-1" OR "PD-L1" OR "Nivolumab" OR "Atezolizumab" OR "Sintilimab" OR "Pembrolizumab" OR "Ipilimumab" OR "Durvalumab" OR "Avelumab")))

**Scopus search strategies**

(TITLE("intratumor* microbio*" OR "intratumor* bacter*" OR "tumor microbio*" OR "tumor bacter*" OR "tumor-resident microbio*" OR "tumor-resident bacter*" OR "tumor-infiltrating bacter*" OR "tumor-infiltrating microbio*") OR ABS("intratumor* microbio*" OR "intratumor* bacter*" OR "tumor microbio*" OR "tumor bacter*" OR "tumor-resident microbio*" OR "tumor-resident bacter*" OR "tumor-infiltrating bacter*" OR "tumor-infiltrating microbio*") OR AUTHKEY("intratumor* microbio*" OR "intratumor* bacter*" OR "tumor microbio*" OR "tumor bacter*" OR "tumor-resident microbio*" OR "tumor-resident bacter*" OR "tumor-infiltrating bacter*" OR "tumor-infiltrating microbio*")) AND (TITLE("immunotherap*" OR "adoptive cell therap*" OR "CAR-T" OR "immune checkpoint blockade*" OR "immune checkpoint inhibitor*" OR "immune modulation" OR "ICI*" OR "CPI" OR "CTLA-4" OR "PD-1" OR "PD-L1" OR "Nivolumab" OR "Atezolizumab" OR "Sintilimab" OR "Pembrolizumab" OR "Ipilimumab" OR "Durvalumab" OR "Avelumab") OR ABS("immunotherap*" OR "adoptive cell therap*" OR "CAR-T" OR "immune checkpoint blockade*" OR "immune checkpoint inhibitor*" OR "immune modulation" OR "ICI*" OR "CPI" OR "CTLA-4" OR "PD-1" OR "PD-L1" OR "Nivolumab" OR "Atezolizumab" OR "Sintilimab" OR "Pembrolizumab" OR "Ipilimumab" OR "Durvalumab" OR "Avelumab") OR AUTHKEY("immunotherap*" OR "adoptive cell therap*" OR "CAR-T" OR "immune checkpoint blockade*" OR "immune checkpoint inhibitor*" OR "immune modulation" OR "ICI*" OR "CPI" OR "CTLA-4" OR "PD-1" OR "PD-L1" OR "Nivolumab" OR "Atezolizumab" OR "Sintilimab" OR "Pembrolizumab" OR "Ipilimumab" OR "Durvalumab" OR "Avelumab"))

**Pubmed search strategies**

("intratumor* microbio*"[Title/Abstract] OR "intratumor* bacter*"[Title/Abstract] OR "tumor microbio*"[Title/Abstract] OR "tumor bacter*"[Title/Abstract] OR "tumor-resident microbio*"[Title/Abstract] OR "tumor-resident bacter*"[Title/Abstract] OR "tumor-infiltrating bacter*"[Title/Abstract] OR "tumor-infiltrating microbio*"[Title/Abstract]) AND ("immunotherap*"[Title/Abstract] OR "adoptive cell therap*"[Title/Abstract] OR "CAR-T"[Title/Abstract] OR "immune checkpoint blockade*"[Title/Abstract] OR "immune checkpoint inhibitor*"[Title/Abstract] OR "immune modulation"[Title/Abstract] OR "ICI*"[Title/Abstract] OR "CPI"[Title/Abstract] OR "CTLA-4"[Title/Abstract] OR "PD-1"[Title/Abstract] OR "PD-L1"[Title/Abstract] OR "Nivolumab"[Title/Abstract] OR "Atezolizumab"[Title/Abstract] OR "Sintilimab"[Title/Abstract] OR "Pembrolizumab"[Title/Abstract] OR "Ipilimumab"[Title/Abstract] OR "Durvalumab"[Title/Abstract] OR "Avelumab"[Title/Abstract])
